# Functional analysis of the cotton *CLE* polypeptide signaling gene family in plant growth and development

**DOI:** 10.1038/s41598-021-84312-8

**Published:** 2021-03-03

**Authors:** Ke Wan, Kening Lu, Mengtao Gao, Ting Zhao, Yuxin He, Dong-Lei Yang, Xiaoyuan Tao, Guosheng Xiong, Xueying Guan

**Affiliations:** 1 State Key Laboratory of Crop Genetics and Germplasm Enhancement, Cotton Hybrid R & D Engineering Center (the Ministry of Education), College of Agriculture, Nanjing Agricultural University, Nanjing, 210095 Jiangsu China; 2 College of Agriculture and Biotechnology, Zhejiang University, Hangzhou, 210058 Zhejiang China

**Keywords:** Molecular biology, Plant sciences

## Abstract

The *CLAVATA3 (CLV3)/EMBRYO SURROUNDING REGION (ESR)–RELATED* (*CLE*) gene family encodes a large number of polypeptide signaling molecules involved in the regulation of shoot apical meristem division and root and vascular bundle development in a variety of plants. *CLE* family genes encode important short peptide hormones; however, the functions of these signaling polypeptides in cotton remain largely unknown. In the current work, we studied the effects of the *CLE* family genes on growth and development in cotton. Based on the presence of a conserved CLE motif of 13 amino acids, 93 genes were characterized as *GhCLE* gene family members, and these were subcategorized into 7 groups. A preliminary analysis of the cotton CLE gene family indicated that the activity of its members tends to be conserved in terms of both the 13-residue conserved domain at the C-terminus and their subcellular localization pattern. Among the 14 tested genes, the ectopic overexpression of *GhCLE5::GFP* partially mimicked the phenotype of the *clv3* mutant in *Arabidopsis*. GhCLE5 could affect the endogenous CLV3 in binding to the receptor complex, comprised of CLV1, CLV2, and CRN, in the yeast two-hybrid assay and split-luciferase assay. Silencing *GhCLE5* in cotton caused a short seedling phenotype. Therefore, we concluded that the cotton *GhCLE* gene family is functionally conserved in apical shoot development regulation. These results indicate that CLE also plays roles in cotton development as a short peptide hormone.

## Introduction

Intercellular communication is essential for the development of tissues and organs. In plants, peptide-receptor signaling modules play an important role in mediating intercellular communication and interactions during development, as well as responding to environmental stimuli^1^. One of the most well explored gene families encoding small peptide ligands is the *CLAVATA3 (CLV3)/EMBRYO SURROUNDING REGION (ESR)–RELATED* (*CLE*) gene family^1–5^. *CLE* gene family peptides are involved in short- and long-distance signal transduction^6,7^.

The WUSCHEL-CLAVATA (WUS-CLV) feedback loop of *Arabidopsis thaliana* maintains stem cell homeostasis in shoot apical meristems (SAMs)^8–10^. Plant SAMs are the sources of all the above-ground parts of the plant^11^. They achieve this by slowly dividing as stem cells and constantly transferring daughter cells to the surrounding marginal area, where they are incorporated into the primordia of the leaves or flowers^12^. In order to maintain the function of SAMs, a balance needs to be struck between the generation of new meristematic cells through division and the separation of cells from the meristem through differentiation^5,9^. The *WUS* encodes a homeodomain transcription factor that actively regulates the activity of meristems and is essential for maintaining stem cell populations during the later stages of embryo development^13,14^. *wus* mutants show SAM growth arrest, and then axillary buds resume growth^14^, showing undulations and phenotypes similar to those in conditions of mild-to-moderate overexpression of *CLV3*^15^. *CLV3* is a member of the *CLE* gene family, which contains a conserved C-terminal CLE motif of 12 to 13 amino acids^16^. The interaction between WUS and CLV3 produces a negative feedback regulation that balances stem cell maintenance and cell differentiation in shoot apical meristems^5,15,17,18^. CLV1 belongs to the leucine-rich repeat–receptor-like kinase (LRR-RLK) subfamily, which contains 21 LRR- and 223 RLK-domain members^19,20^. One of the differences between CLV1 and CLV2 is that there is no kinase domain in CLV2^19,21,22^. CLV1 homodimers play a role in parallel to the CLV2-CORYNE heterodimer, in which CLV2, with its extracellular domain, interacts with CORYNE, which has an intracellular kinase domain to transduce the CLV3 signal^23,24^.

In addition to maintain the homeostats of SAM, other *CLE* gene family members also play important roles in multiple plant organ development control^25^. CLE14 is one of the main regulator of root apical meristem differentiation^26^. CLE9/10 and CLE25 plays roles both in xylem formation and stomatal development^27,28^. CLE19 is reported to regulate the cotyledon and endosperm development in *Arabidopsis*^29^. Two *Lotus japonicus* CLE peptides are reported to be involved with nodule nodule organ formation^30^. A homolog of AtCLV3 in *Brassica napa* controls the multilocular silique development^31^.

Cotton (*Gossypium*) is one of the most important cash crops and provides fiber for the textile industry. Cotton is also a model polyploid plant used to study whole-genome duplication events^32^. GhWUS is an important regulator of somatic embryogenesis and bud regeneration^33^. Previously, 55 *CLE* genes were extracted from the close D_5_ donor genome of allotetraploid cotton species, wild diploid cotton *Gossypium raimondii*^2^. The role of *CLE* genes in the upland cotton cultivar is still unknown, however. In the present study, we carried out a functional analysis of *CLE* genes in the upland cotton (*Gossypium hirsutum*) genome. By means of a genome-wide study, translational analysis, protein interaction assay, and virus-induced gene silencing (VIGS) technology, we identified members of the cotton *CLE* gene and screened out the cotton GhCLE5 with ectopic effects in the reassembly of the *Atclv3* mutant.

## Results

### The *CLE* gene family in upland cotton

**Figure 1:**
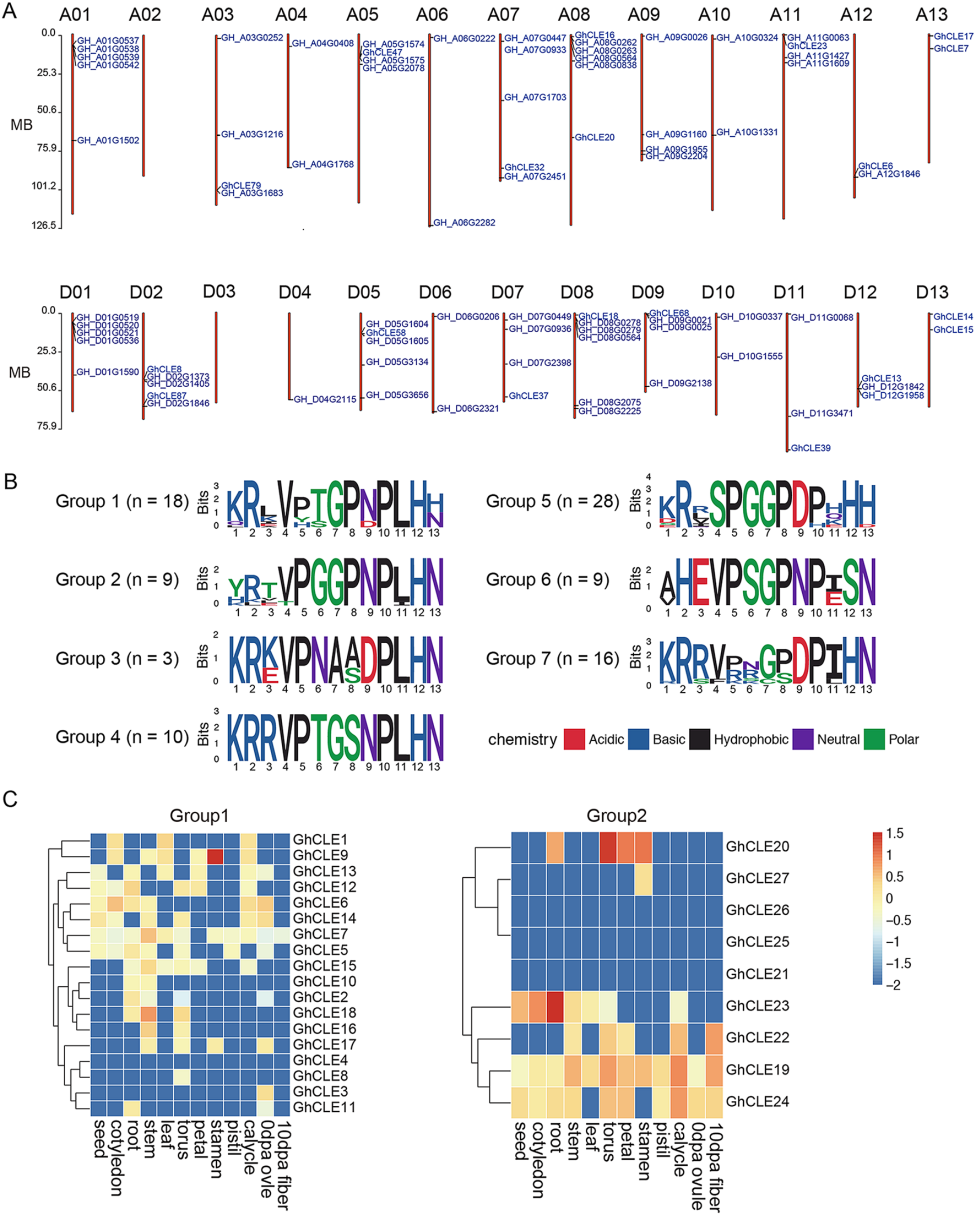
The *GhCLE* gene family in the upland cotton genome. (**A**) The chromosome distribution of *GhCLE* genes. A01-13 and D01-13 indicate the chromosome number of upland cotton. The *GhCLE* genes are labeled on the relative locations on the corresponding chromosome. (**B**) The sequence logos of CLE motif peptides for seven *GhCLE* gene family groups. (**C**) Heat maps representing the Group 1 and Group 2 *GhCLE* gene expression patterns in the cotton seed, cotyledon, root, leaf, torus, petal, stamen, pistil, calycle, and ovule tissues 0 day post anthesis (0 DPA) and in 10 DPA fibers.

Based on the sequences of *CLE* genes in the *Arabidopsis* genome, homologous alignment of the psi-BLAST and *G. hirsutum* Texas Marker-1 (TM-1) genome sequences was performed. In total, 93 genes containing the CLE motif were obtained and named sequentially according to their distribution on the Upland cotton TM-1 chromosomes (Fig. 1A).

Forty-two genes were in the A_T_ subgroup of *G. hirsutum* and 42 were in the D_T_ subgroup (Fig. 1A, Supplemental Table 1). The distribution map showed that chromosomes A02 and ChrD03 had no *CLE* genes. The *CLE* genes were evenly distributed on the other chromosomes. Most of the *CLE* genes were composed of a single exon (Supplemental Figure 1). Through collinear analysis, we identified a total of 35 pairs of homeologous genes in the A and D subgenomes (Supplemental Table 2). The average Ka/Ks value was 0.54, and the Ka/Ks values of three pairs of direct homologous genes exceeded 1 (Supplemental Table 2), indicating that the *CLE* gene family had a relatively fast evolutionary speed.

The *CLE* family members have a highly conserved amino acid sequence containing 12 to 13 amino acids, named the CLE motif. Previous studies in the model plant *Arabidopsis* have reported that *CLE* family members can be classified into four or five groups according to the diversity of their CLE motif sequences, which represent unique biologic functions^34^. We adapted the analysis of the CLE motifs and classified the cotton *CLE*s into seven categories, named Groups 1 to 7 (Fig. 1B, Supplemental Figures 2 and 3). The CLE motif of Group 1 showed the highest sequence similarity to that of the CLV3 motif. That of Group 6 showed the highest similarity to the B-type TDIF CLE^35^.

**Figure 2:**
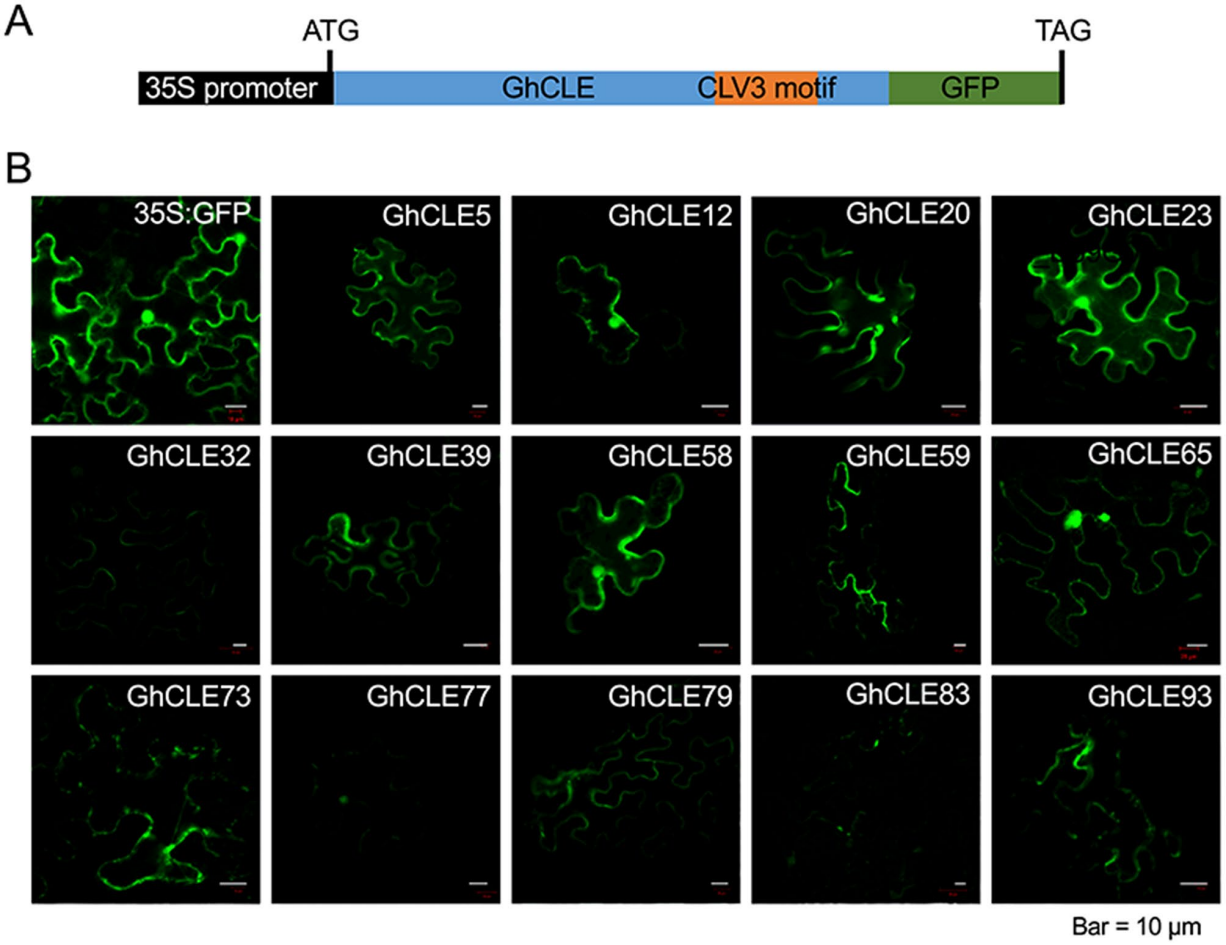
GFP fusion signal illustrating the subcellular localization of the selected *GhCLE* genes from seven groups using transient expression in tobacco leaves. (**A**) Schematic chart showing the construct structure of the *35S::GhCLE::GFP* vector. (**B**) *GhCLEs::GFP* confocal micrographs at 488 nm excitation. Each examination included five biologic replications.

**Figure 3:**
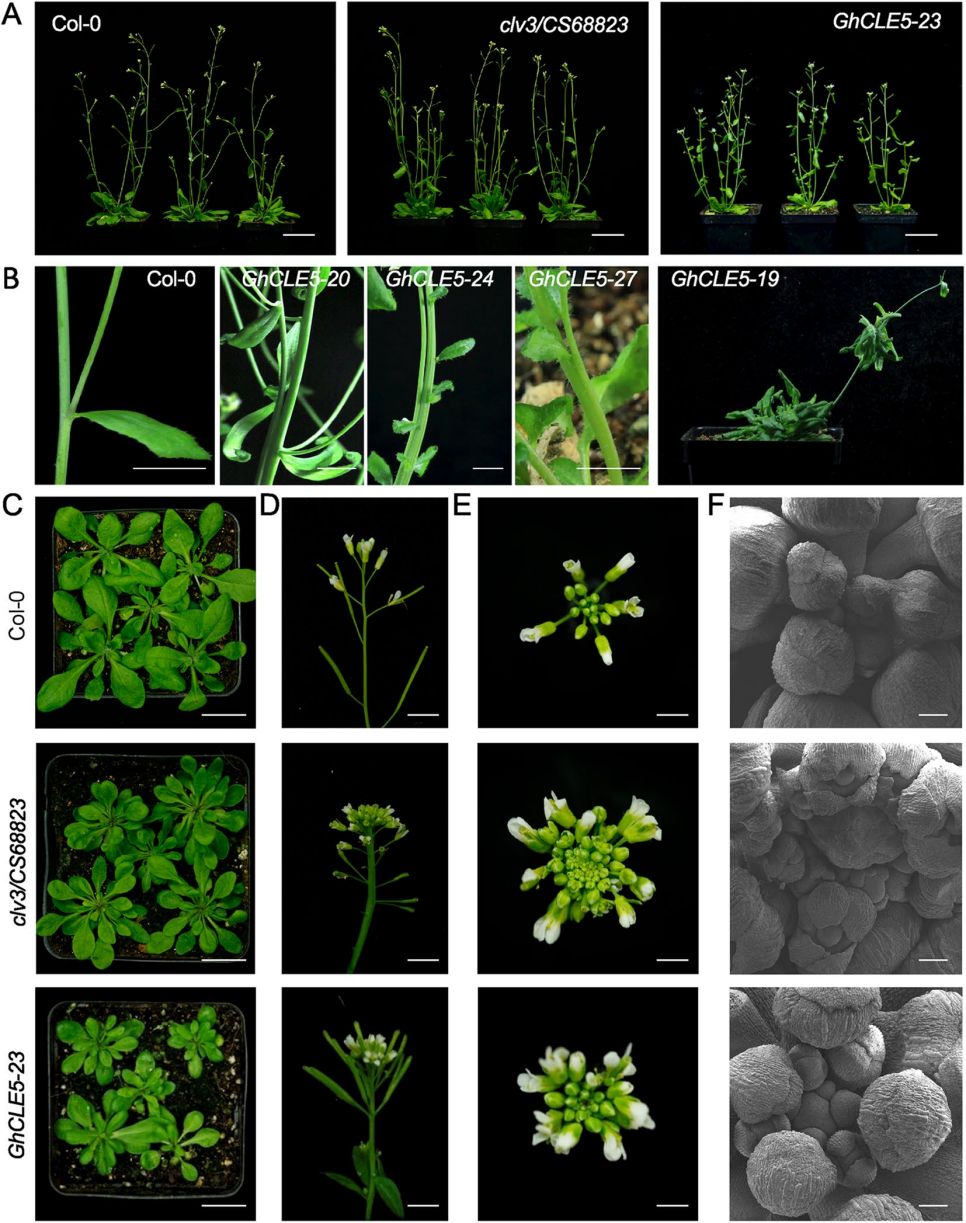
The phenotype of *Arabidopsis thaliana* with ectopic expression of *GhCLE5*. (**A**) The 6-week-old plant. (**B**) The stems of the *GhCLE5* transgenic *Arabidopsis* plants from multiple T1 lines. (**C**) The photo images of 4-week-old plants. Bar = 2 cm. (**D**) The vertical view of florescence. Bar = 5 mm. (**E**) The bird’s-eye view of the florescence. Bar = 1 mm. F: The scanning electron microscope (SEM) images of the shoot apical meristem (SAM). Bar = 100 μm.

The seven *GhCLE* groups showed distinct expression patterns in the cotton root, cotyledon, leaf, stem, torus, petal, stamen, pistil, calyx, ovule, and fiber tissues. Groups 1, 2, 6, and 7 showed ubiquitous expression patterns in most of the cotton tissues (Fig. 1C, Supplemental Figure 4). Genes in Group 3 were predominantly expressed in the hypocotyl and cotyledons of seedlings and stems (Supplemental Figure 4), whereas genes in Group 5 were specifically expressed in flower tissues, including torus, petal, and stamen tissues (Supplemental Figure 4). Some members of Groups 2 and 5 had consistently low expression patterns in most of the cotton tissues; this could indicate that these are stress-responsive genes.

**Figure 4:**
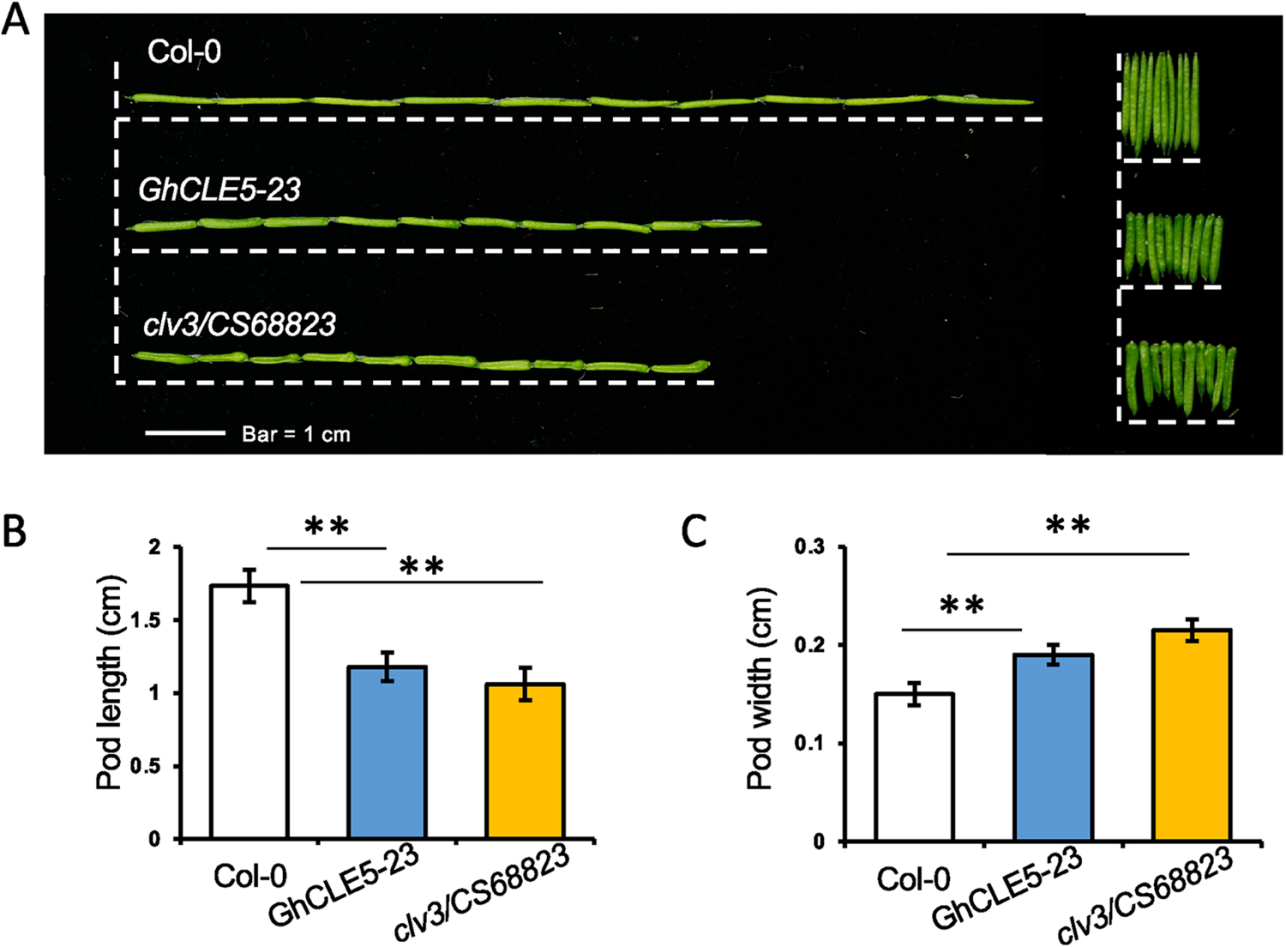
The phenotypes of *GhCLE5* transgenic *Arabidopsis* siliques. (**A**) The photo images of the siliques. The histogram of the silique pod length (**B**) and width (**C**). N = 30. **, *p* < 0.01; Student’s *t* test. Error bar = Std.

To validate the CLE genes characterized in allotetraploid upland cotton of *G. hirsutum*, we further performed a comparison between the CLEs from the D subgenome in allotetraploid (Gh) and diploid D genome (Gr) previously reported^2^ (Supplement Table 3). Of the 55 Gr-genome CLE genes reported by Goad et al., 42 orthologs are found among our Gh *CLE* genes. In addition, 10 *CLE* genes from Goad are not present in the D subgenome of Gh, and another 3 *CLE* genes from Gr only have orthologs in the A subgenome of Gh. Finally, 2 *CLE* genes from the D subgenome in our report have not been identified previously in Gr. These results validate the *CLE* genes we characterized in Gh and also imply the dynamics of *CLE* gene evolution after polyploidization in allotetraploid species.

### Unprocessed GhCLE proteins were predominantly localized to the cell perimeter

To investigate the translational capacity of the cotton *CLE* genes, we performed subcellular localization tests. Two candidate genes from each cotton *CLE* group on average (Supplemental Table 4) with relatively high expression activity were selected for this study. GFP was fused to the 3′ end of the candidate genes with a 35S promoter (Fig. 2A). The GFP fusion constructs were transiently expressed in tobacco leaves, and fluorescence was observed. Six proteins, GhCLE12, GhCLE20, GhCLE23, GhCLE58, and GhCLE65, GhCLE77 were located in the cell membrane and nucleus (Fig. 2B). Eight proteins, GhCLE5, GhCLE32, GhCLE39, GhCLE59, GhCLE73, GhCLE79, GhCLE83, and GhCLE93, were located in the cell membrane (Fig. 2B). Previous studies have shown that the CLE proteins can be processed into short peptides that can be secreted across the cell membrane. The *GhCLEs::GFP* fusion signal represented the proteins after translation but before they were processed. In Fig. 2, cotton CLE proteins with a GFP signal can be seen to be predominantly distributed in the cell membrane regions in comparison with the *35S::GFP* signal. Although the GFP fused construct might affect the CLE protein process to some extent, the *CLE::GFP* localization exhibited a unique pattern distinct from the GFP itself, which could be a ready-to-go position for the processed peptide to undergo transmembrane movement. These subcellular localization signals indicate that the cotton CLEs are highly likely to be secretory proteins similar to those found in *Arabidopsis*.

### Ectopic overexpression of *GhCLE5* mimicked the *Atclv3* phenotype

The 14 selected *GhCLE* candidate genes were transformed into the wild type of *A. thaliana* Col-0 for further functional study. The ectopic expression of *GhCLE5* caused the highest variation in phenotype in the *Arabidopsis* transgenic lines. Among the 34 T_1_ positive lines of *35S::GhCLE5* transgenic *Arabidopsis* (Supplemental Figures 5 and 6), 12 showed phenotypes in the shoot that clearly differed from the wild type (Fig. 3, Supplemental Table 5). The phenotypic traits included dwarf plants, *clv3*-like florescence, fasciated stems, and club-shaped siliques (Fig. 3B to F, Supplemental Table 5). A representative line, *GhCLE5-23*, is shown in Fig. 3A,C, with curved rosette leaves and bushy stems in the bolting plants. The siliques were shorter than the wild type (Col-0) but not as short as *clv3* (Fig. 4A–C)*. GhCLE5-23* exhibited florescence similar to that of *clv3* (Fig. 3D,E). The flower primordium and floral bud number were obviously more in *Atclv3* and GhCLE5 transgenic lines than that of Col-0 (Fig. 3F). Other lines, such as *GhCLE5-10, GhCLE5-20*, *GhCLE5-24*, and *GhCLE5-27*, showed fasciated stems (Fig. 3B, Supplemental Table 5). *GhCLE5-19* showed an extreme phenotype of rosette leaf regeneration on the stem (Fig. 3B) with infertile flowers. *35S::GhCLE5* did not have any consistent impact on the root growth of the transgenic lines (Supplemental Figures 7 and 8).

**Figure 5:**
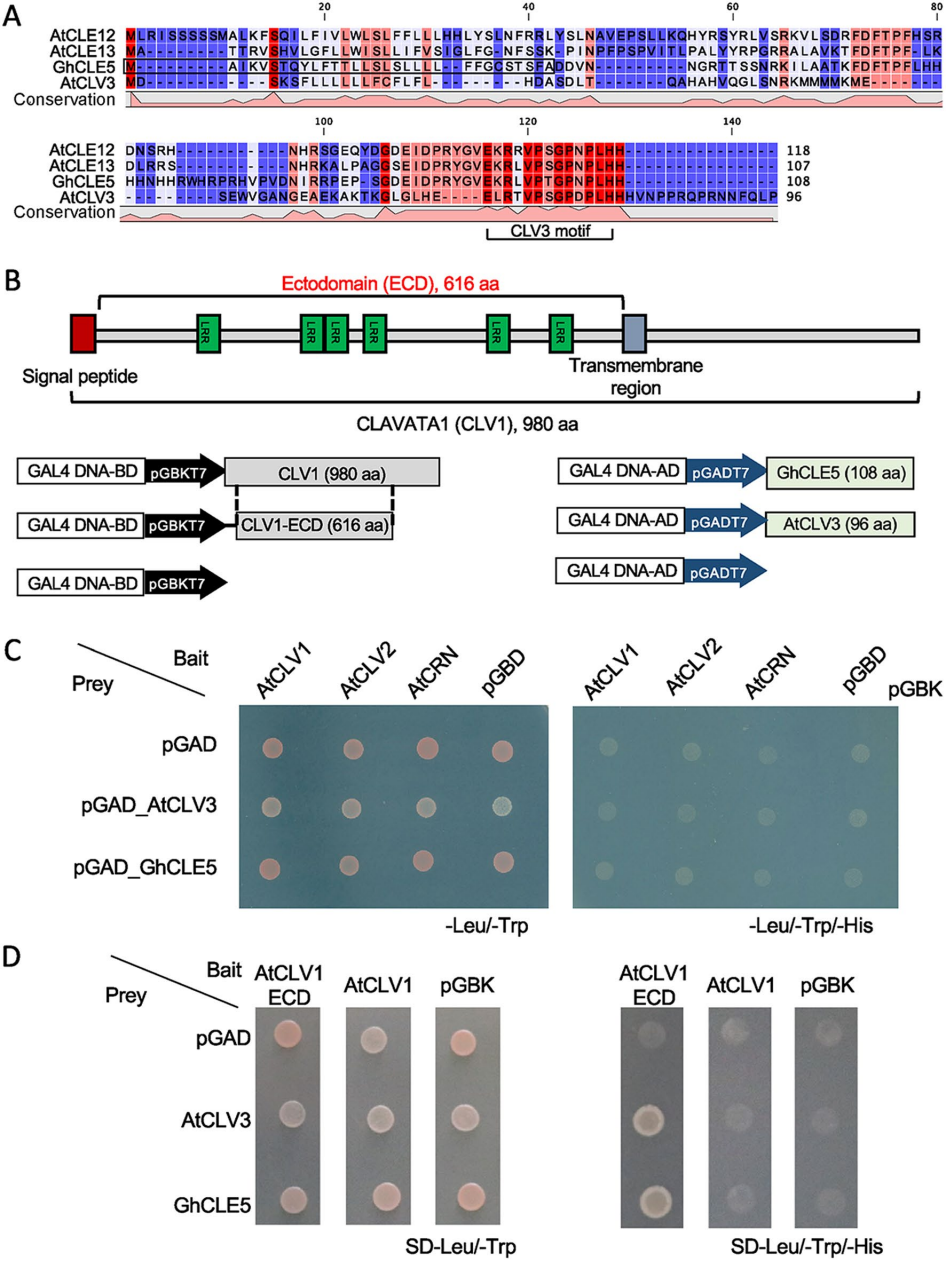
The interaction between AtCLV1, AtCLV3, and GhCLE5. (**A**) The amino acid sequence alignment of GhCLE5, AtCLE12, AtCLE13, and AtCLV3. The conserved CLV3 motif is indicated. The black box is the putative signal peptide (http://smart.embl-heidelberg.de/). (**B**) The schematic chart showing the protein structure of AtCLV1 and the design for the yeast two-hybrid assay. (**C**) and (**D**) Photo images for the yeast two-hybrid assay that tested the interaction between AtCLV3, GhCLE5, and AtCLV1.

**Figure 6:**
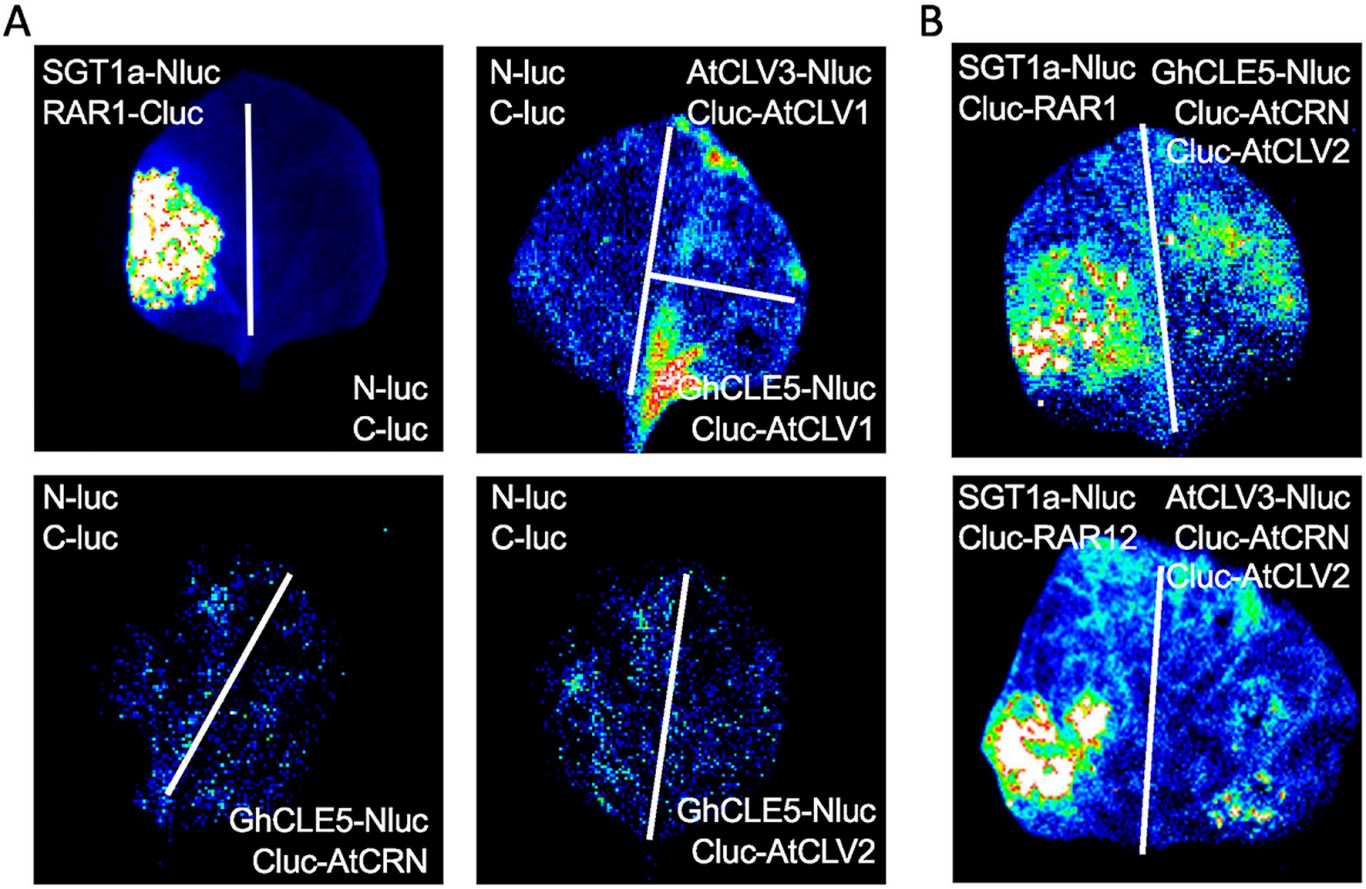
Validation of GhCLE5 binding activity with the CLV1, CLV2, CRN, and CLV2/CRN heterodimers. (**A**) The split-luciferase assay to detect the GhCLE5 binding activity with AtCLV1, AtCLV2, and AtCRN, respectively. The SGT1a-Nluc and RAR1-Cluc are used as positive controls for the binding protein. (**B**) The split-luciferase assay to detect the GhCLE5 binding activity in parallel with the AtCLV2 and AtCRN together. Each examination included three biologic replications.

**Figure 7:**
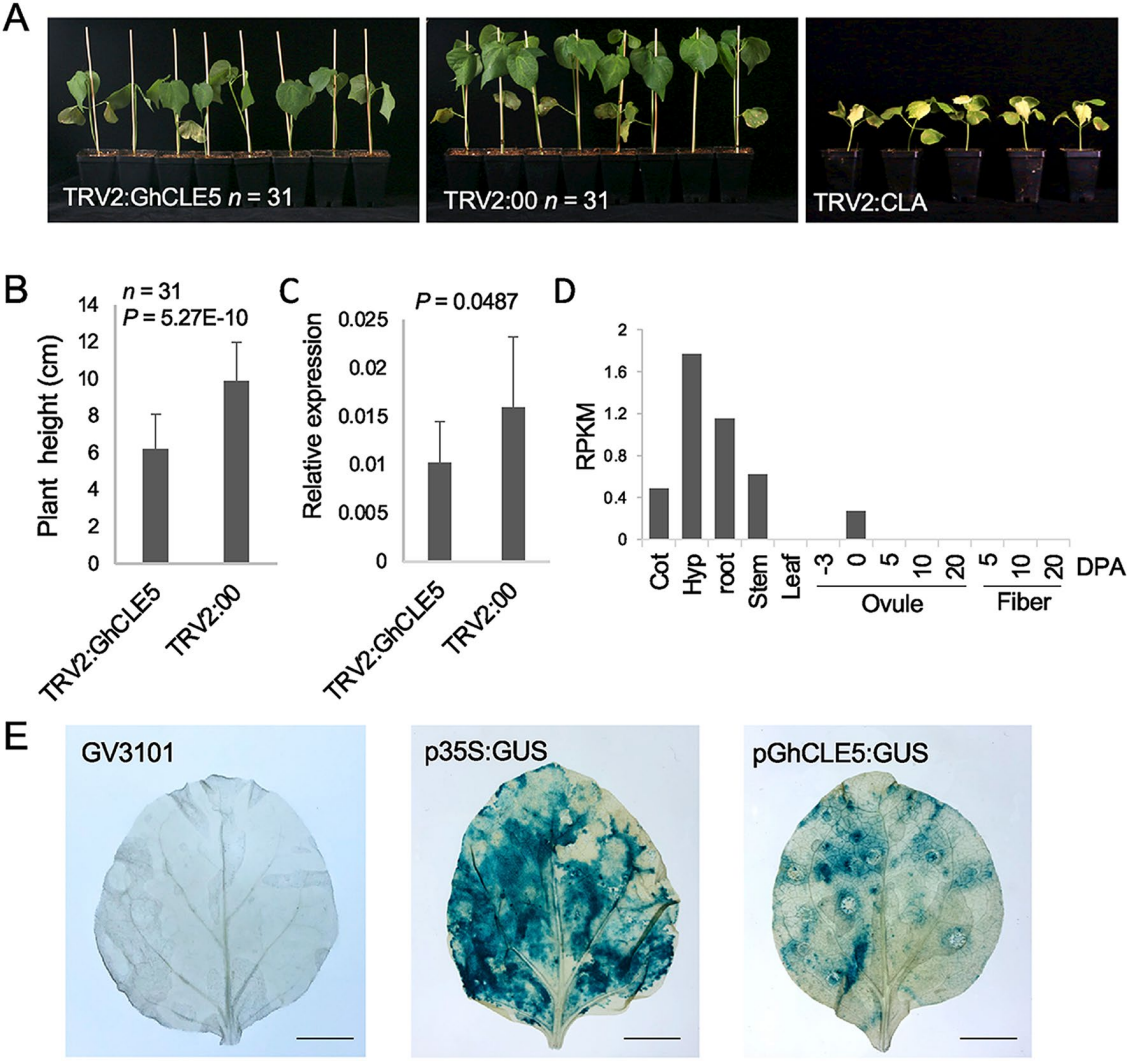
The phenotype of cotton seedlings following VIGS to suppress the expression of *GhCLE5*. (**A**) VIGS-treated cotton seedlings. The population sizes of the *pTRV2:GhCLE5* and *pTRV2:00* treatments were both 31. The *pTRV2:CLA* was the positive control group for VIGS showing bleached leaves. (**B**) Histogram indicating the differences in plant height between the *pTRV2:GhCLE5* and *pTRV2:00* groups derived from the VIGS-treated plants shown in panel A (Student’s *t* test). Error bar = Std. (**C**) Histogram showing the relative expression of *GhCLE5* in the *pTRV2:GhCLE5* and *pTRV2:00* groups derived from the VIGS-treated leaf tissues shown in panel A (Student’s *t* test). Error bar = Std. (**D**) Histogram showing the *GhCLE5* expression pattern in the cotyledon (Cot), hypocotyl (Hyp), root, stem, leaf, ovule, and fiber tissues of upland cotton using RNA-seq RPKM values. DPA, days post anthesis. (**E**) The imagines show the GUS staining of the tobacco leaves transient expressed with the GUS reporter driven by *GhCLE* promoter (pGhCLE5:GUS). The negative control is GV3101 bacterial injection buffer with and without the pBI121 vector containing a GUS driven by 35S promoter (p35S:GUS). Three biological replicates were applied for each test group. Scale bar = 1 cm.

**Figure 8:**
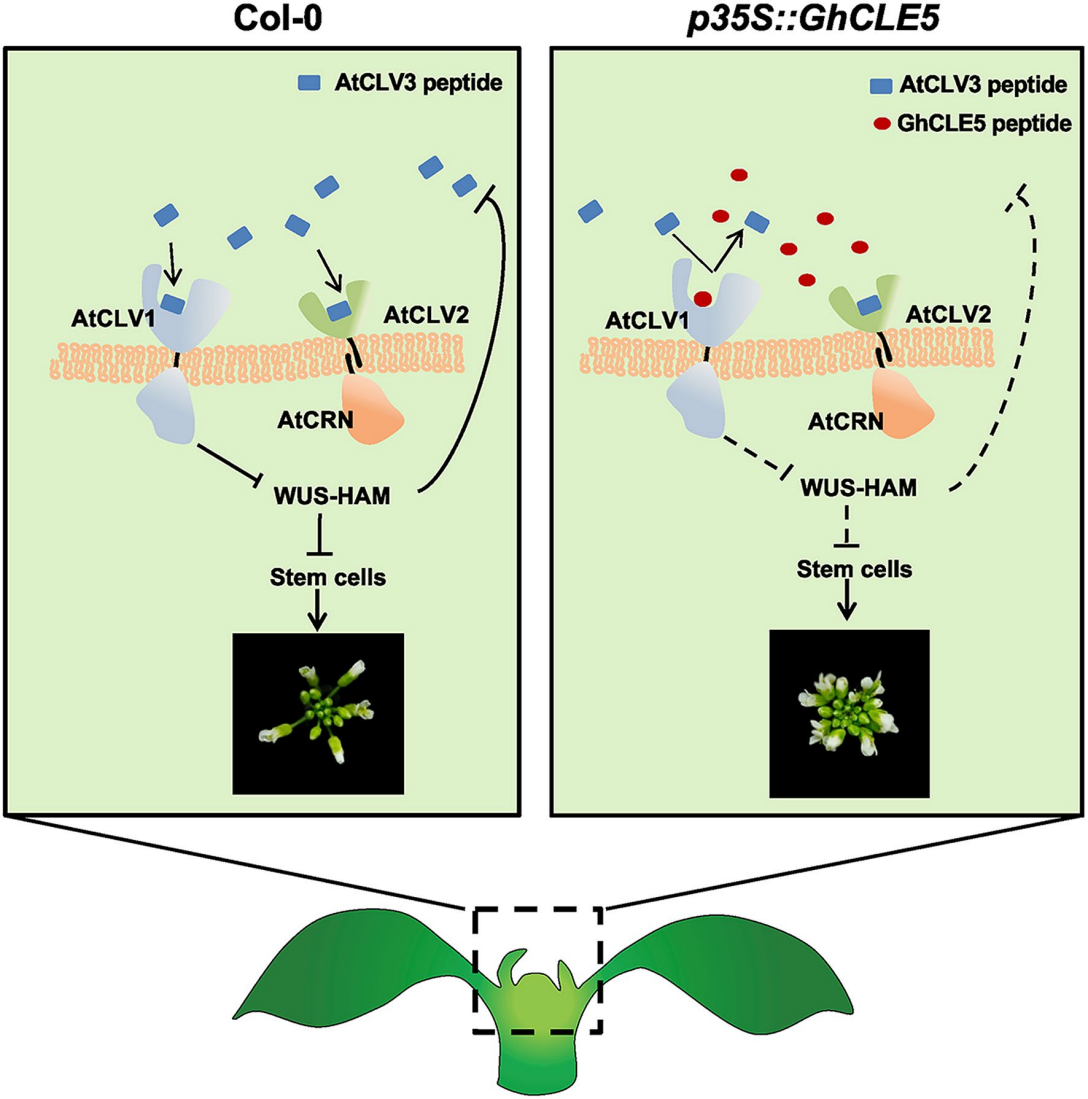
The working model for the ectopic effects of GhCLE5 in *Arabidopsis*.

The phenotypes observed in the *35S::GhCLE5* transgenic lines partially resembled the *Atclv3* phenotype in terms of the morphology of the florescence and fasciated stems. AtCLE12/13 was reported to lead to a dwarf plant with short silique, which mimics the *Atclv3* phenotype at overexpression^34,36^. The ectopic expression of AtCLE1-7 showed almost identical phenotype with *Atclv3*^37^*.* The amino acid alignment shows that the CLE motif of GhCLE5 is similar to AtCLE12, AtCLE13, and AtCLV3 (Fig. 5A). Therefore, the ectopic expression of *GhCLE5* interrupted the AtCLV3 function in a way that may be similar to the ectopic expression of AtCLE12. We speculated that GhCLE5 could behave as a signal either by competing with the AtCLV3 peptide or blocking the downstream signaling transduction.

### GhCLE5 interacted with the CLV3 receptor complex

GhCLE5 contained a predicted signal peptide on the N-terminus (http://smart.embl-heidelberg.de/) (Fig. 5A). The active translation of *GhCLE5* was further confirmed by GFP signal examination (Supplemental Figure 6B). *GhCLE5::GFP* signals were observed in root, leaf, and stem tissues (Supplemental Figure 6B). As previously reported, the CLE proteins must be processed into short peptides containing the CLE motif before they carry out their function. Therefore, the *GhCLE5::GFP* proteins observed were unprocessed proteins with no function. However, the GhCLE5::GFP signal indicated that GhCLE5 is highly likely to be a transmembrane protein that is ready to be digested and secreted across the cell membrane. If GhCLE5 was processed into a peptide to occupy the position of the CLV3 motif as a hormonal molecule, the *GhCLE5::GFP* protein should have undergone protein processing and the GFP degradation would render it undetectable. Furthermore, we did not observe any GFP signal in the SAM tissue of any of the phenotypic transgenic *GhCLE5::GFP* plants. Therefore, we speculate that GhCLE5 is processed into a short peptide that could compete with the CLV3 motif in SAM development regulation.

To determine how the ectopic overexpression of *GhCLE5* in *Arabidopsis* led to a phenotype partially resembling the *Atclv3* phenotype, we further examined the binding capacity of GhCLE5 in each of the receptor complexes of CLV1, CLV2, and CRN/CLV2. AtCLV1 contains an ectodomain (ECD) on the N-terminus and a transmembrane domain on the C-terminus (Fig. 5B). A yeast two-hybrid assay was first conducted to detect the binding efficiency of GhCLE5 with the whole length of AtCLV3 receptors, AtCLV1, AtCLV2, and AtCRN. The assay did not show any direct interaction between GhCLE5 and AtCLV1, AtCLV2, or AtCRN (Fig. 5C). There was also no interaction observed between AtCLV3 and any of these proteins. This may be because the yeast system failed when used on proteins with a transmembrane structure. When we used the ECD of AtCLV1 on the pGBK, the yeast two-hybrid result showed clear binding of GhCLE5 and the ECD domain of AtCLV1(Fig. 5D). To confirm the binding capability between GhCLE5 and the AtCLV3 receptors, split-luciferase assays were performed. These assays no interactions between GhCLE5-Nluc and either Cluc-AtCLV2 or Cluc-AtCRN were detectable (Fig. 6A). However, there showed an interaction between GhCLE5-Nluc and Cluc-AtCLV1 (Fig. 6B). CLV3/CLV2/CRN can form a complex that regulates the SAM status in *Arabidopsis*. We hypothesize that GhCLE5 could replace CLV3 to form a complex with CLV2 and CRN.

### *GhCLE5* functional validation in cotton seedling height regulation

To investigate the function of *GhCLE5* in cotton, we employed VIGS technology in upland cotton accession TM-1. The *GhCLE5*-silenced plants showed relatively slow development in terms of seedling height (Fig. 7A–C). The expression of *GhCLE5* RNA was relatively high in the hypocotyl, root, and stem tissues of cotton seedlings (Fig. 7D). The promoter activity of *GhCLE5* was validated by the transient expression assay using tobacco leaf assay (Fig. 7E). The expression pattern also supported a functional role for *GhCLE5* in seedling height control. Plant height is under the regulation of multiple factors, including, but not limited to, the development of vascular tissue and the SAM status. Therefore, the phenotypes of ectopic *GhCLE5* expression in *Arabidopsis* support the proposed endogenous function of *GhCLE5* in cotton seedlings.

## Discussion

Based on the presented data, we propose that GhCLE5 could occupy the CLV3 motif binding sites with CLV1, CLV2, and CRN in the *Arabidopsis* ectopic overexpression line (Fig. 8). Due to the sequence similarity of CLE motifs, CLV3 receptors can be engaged with ectopic GhCLE5. In this way, the signal mediated by the CLV3/CLV1 and CLV3/CLV2/CRN complexes could not be transmitted. The *Arabidopsis* ectopic GhCLE5 transgenic lines might have unstable protein-processing efficiency, because the ectopic expression of GhCLE5 led to a variety of developmental phenotypes, each of which partially mimicked the *Atclv3* phenotype. This also indicates that the cotton *GhCLE* gene family could be a potential functional regulator for plant development.

*CLE* genes can derive short peptides that function as hormone-signaling molecules to direct the development of plant shoots and roots. The *CLE* gene sequences vary greatly from species to species. The conserved CLE motif is as short as 12 to 13 amino acids, which makes it difficult to identify *CLE* gene family members in alternative genomes. Cotton is a fiber plant and also a model plant for polyploid genome study. We identified 93 *CLE* genes in the upland cotton genome, with more than 40 members from each subgenome. This number is comparable with the CLE gene members identified in *G. raimondii* genome (55)^2^. The gene number is not significantly larger than that in *Arabidopsis* (45), although the cotton genome is about 10 times larger than that of *Arabidopsis*, due to multiple whole-genome duplication events^38^. The *CLE* gene family was not enlarged together with the whole-genome duplication events, which indicates its importance and the strength of the selection pressure on it.

Although the *CLE* gene family was not enlarged in cotton, the CLE motif was profoundly diversified. According to the CLE motif sequence, new groups of CLE patterns can be distinguished. However, no identical CLE motif of CLV3 was detected in the cotton genome. In addition, the homologous *WUS* gene in cotton is known to play a similar role in embryotic callus induction^33,39,40^. Given the conserved circuit of SAM maintenance, it seems that cotton has derived a unique CLE motif to fulfill this function. Here we demonstrated that GhCLE5 can interact with the CLV3 receptors in tobacco leaves. However, the main function of *GhCLE5* in cotton is largely unknown. VIGS treatment did not have a dramatic impact on cotton SAMs; however, the efficiency of VIGS was not high. We cannot rule out the possibility that any other GhCLE member could also be involved at this stage, especially those from Group I. The endogenous receptors of GhCLE5 in cotton are still unknown.

GhCLE Might Undergo Protein Processing. Protein processing is critical for CLE proteins. A typical CLE protein is located on the membrane with a signal peptide on the extracellular surface. The CLE motif can be processed into a peptide that serves as a signaling molecule. The cellular localization assay demonstrated that cotton CLEs are predominantly located on the cell membrane. According to the predicted protein structure, GhCLE5 contains a signal peptide on the N-terminal. The C-terminal with the CLE motif is also predicted to be an outside arm. In *35S::GhCLE5::GFP* transgenic *Arabidopsis*, GFP signals represented unprocessed or incompletely processed proteins. The GhCLE5-23 and GhCLE5-19 lines, which exhibited a strong phenotype, had weak GFP signals and strong RNA transcription activity. Statistical analysis was not conducted due to the very limited biologic replication numbers. However, observation indicated that the CLE5 processing efficiency could be high in those lines. We can take this as an indication that cotton employs the conserved short peptide–processing mechanism of CLE proteins.

## Materials and methods

### Retrieval of the *CLE* gene in upland cotton

The reference genome and annotation data of the upland cotton *G. hirsutum* were retrieved from the Cotton Research Institute, Nanjing Agricultural University (http://mascotton.njau.edu.cn/index.htm)^41^. The amino acid sequence of the *Arabidopsis CLE* gene was retrieved from the TAIR database (http://www.arabidopsis.org/). To identify *CLE* gene candidates in cotton, the *Arabidopsis CLE* genes were used as a query source for searching against the cotton genome using BLASTP^41^. Only genes with translated sequences with a CLE motif, signaling peptides in the N-terminal, and a molecular weight of less than 15 KD were considered as cotton *CLE* genes^42^.

### Evolutionary analysis and structure prediction

The screened CLE protein sequences were aligned using ClustalX (http://www.clustal.org/) with default parameters. In constructing the phylogenetic tree, ModelGenerator (http://mcinerneylab.com/software/modelgenerator/) software^43^ was used to calculate the best alternative model. PhyML (http://www.atgc-montpellier.fr/phyml/) was used to construct the phylogenetic tree with the parameters Bootstrap100 and model JTT. A Gene Structure Display Server (GSDS, http://gsds.gao-lab.org/) was used to analyze the intron–exon structure of genes.

### Chromosome mapping

According to the genomic annotation file, the *CLE* genes were tagged on the chromosome using the R package chromPlot (http://www.bioconductor.org/packages/release/bioc/html/chromPlot.html)^44^. Orthologous *CLE* genes and duplicated *CLE* genes were identified using MCScanX (http://chibba.pgml.uga.edu/mcscan2/) software with default parameters^45^.

### *CLE* gene classification

The hormonal fragment of CLE proteins is a conserved 13-amino-acid sequence, namely, the CLE motif. CLAN software (https://omictools.com/clans-tool) was used to cluster the *CLE* genes based on the similarity of their translated CLE motifs. Next, *CLE* genes were clustered into seven groups, and the R package ggseqlogo^46^ was used for visualization of the motif of each group.

### *CLE* gene expression analysis

RNA-seq data from cotton tissues (root, cotyledon, leaf, stem, torus, petal, stamen, pistil, calyx, ovule, and fiber) were downloaded from our previously released publication^47,48^ and data (PRJNA248163, PRJNA490626). Raw data were trimmed using Trimmomatic (http://www.usadellab.org/cms/?page=trimmomatic) software^49^. Data on the expression of genes were normalized into FPKM using TopHat2 and Cufflinks^50^. Next, the expression levels of *CLE* genes were visualized using the R language package Pheatmap (https://cran.r-project.org/web/packages/pheatmap/).

### Vector construction and plant transformation

The total RNA was isolated from the Upland cotton (*G. hirsutum*) leaf samples using the Rapid RNA Extraction Kit (Zhong Ding Biology, RK2002). Full-length cDNA was synthesized using HiScript II Q RT SuperMix (Vazyme, R222-01) for qPCR. To generate *GhCLE* overexpression plants using *A. thaliana* Col-0 as a background, the full-length coding sequences of *GhCLEs* were first amplified using the primers listed in Supplemental Table 6, and then cloned into the cloning vector *pBINPLUS.GFP4* via *Sal* I and *Bam*H I restriction enzyme sites. *Arabidopsis* transformation was performed using a floral dipping protocol with the *Agrobacterium tumefaciens* GV3101 strain.

### RT-PCR

Primer Premier (Primer Premier 5 1.0, https://macdownload.informer.com/primer-premier/) software was used to design RT-PCR primers (Supplemental Table 6) based on the sequences of the *GhCLE5* genes. The *A. thaliana* reference gene was *UBQ5,* and the *G. hirsutum* reference gene was *histone 3*. Nanjing Prime Biotech Co., Ltd., performed the primer synthesis. Using the SYBR Green I dye method, the 20 μL reaction system in the PCR tube was mixed with 0.5 μL of each of the left and right primers, 1.5 μL of the cDNA template, and 7.5 μL of the ddH_2_O. RT-PCR was carried out using a Roche LightCycler 480 real-time PCR instrument. Expression level analysis was performed in triplicate using the minimum number of sample threshold cycles (Ct value) and 2^−ΔΔCt^ methods.

### Subcellular localization

Subcellular localization was performed by cloning the full length of the *GhCLE* coding sequences (Supplemental Table 6) into the *pBINPLUS.GFP4* vector. The genes were fused in-frame with green fluorescent protein (GFP) for expression under the control of the CaMV35S promoter. The fused *35S::GhCLEs::GFP* construct was transformed into tobacco (*Nicotiana benthamiana*) by infiltration with the *A. tumefaciens* GV3101 strain, and GFP signals were observed using a laser confocal microscope (Zeiss LSM780) with 488-nm excitation.

### Scanning electron microscopy (SEM)

The shoot apical tissue of 5-week old *Arabidopsis* were harvested for the SEM sample preparation. The sample was first fixed with 2.5% glutaraldehyde in phosphate buffer (0.1 M, pH7.0) for more than 4 h; washed three times in the phosphate buffer (0.1 M, pH7.0) for 15 min at each step; then post fixed with 1% OsO_4_ in phosphate buffer for 1–2 h and washed three times in the phosphate buffer (0.1 M, pH7.0) for 15 min at each step. The sample was first dehydrated by a graded series of ethanol (30%, 50%, 70%, 80%, 90% and 95%) for about 15 min at each step, then dehydrated two times by alcohol for 20 min at each step or stored in alcohol. The sample was dehydrated in Hitachi Model HCP-2 critical point dryer. The dehydrated sample was coated with gold–palladium in Hitachi Model E-1010 ion sputter for 4–5 min and observed in Hitachi Model SU-8010 SEM.

### The application of synthetic peptides to *Arabidopsis*

The CLE motif of GhCLE5 was selected for chemical synthesis (GL Biochem Ltd., Shanghai, China) (RLVPTGPNPLHH, purity: 94.1%, 10 mg/tube). The synthesized peptides were fully dissolved with ddH_2_O. After complete dissolution, this stock buffer was filtered and sterilized with a 0.22 μm sterilizer. The 1/2MS medium was sterilized by autoclaving for 15 min, cooled, and then different amounts of peptide stock buffer were added to configure plates with the concentration gradient assigned in the corresponding tests. The five treatments were 0 nmol/L, 10 nmol/L, 10^2^ nmol/L, 10^3^ nmol/L, and 10^4^ nmol/L. *Arabidopsis* seeds were cultivated under a 16/8 h light and dark cycle. Root lengths were examined and photographed at two weeks after germination.

### Yeast two-hybrid assay

The full-length coding sequences of *AtCLV2*, *AtCLV1, AtCRN* and *AtCLV1-ECD* were each fused to the Gal4 DNA binding domain of *pGBKT7*. The full-length coding sequences of *AtCLV3* and *GhCLE5* were each fused to the Gal4 activation domain in *pGADT7*. The constructed bait and prey composition was co-transformed into the yeast strain gold yeast. Two days after growth on SD-Leu/-Trp plates, the interaction between the bait and the prey was observed on the SD-Leu/-Trp/-His selective medium. Yeast strains containing *pGBKT7_AtCLV3* and *pGBKT7_GhCLE5* and a negative *pGADT7* vector were used as negative controls. The constructed bait and prey composition was co-transformed into the yeast strain gold yeast following the kit manufacturer’s instructions (Frozen-EZ Yeast Transformation II Kit, Zymo Research). All primer information can be found in Supplemental Table 6.

### Luciferase complementation for protein–protein interactions

We adapted a split-luciferase assay method^51^ to determine the protein–protein interactions. The full-length coding sequences of *AtCLV2*, *AtCLV1,* and *AtCRN* were each fused to *pCAMBIA1300-cLUC*. The full-length coding sequences of *AtCLV3* and *GhCLE5* were each fused to *pCAMBIA1300-nLUC*. The fused constructs were transformed into tobacco (*N. benthamiana*) by infiltration with the *A. tumefaciens* GV3101 strain. The *Agrobacterium* was shaken overnight on a shaker at 28 °C until the bacterial solution turned orange–yellow. Following overnight culture, the solution was centrifuged at 4000 rpm for 10 min, and the cells were collected. The inoculation dye solution was resuspended to an OD_600_ of 1. The prepared bacterial solution was allowed to stand in the incubator at 28 °C for 2 h in the dark. The N-LUC and C-LUC solutions were mixed at a ratio of 1:1 and then injected into the back of the tobacco leaves. The tobacco plants were covered with a black plastic bag to prevent direct light from reaching them and were placed in a light incubator at 23 °C for 48 h. After the dark treatment, the black plastic bag was removed, and the tobacco plants were placed in a light incubator at 28 °C. After 16 h of light exposure, the LUC activity was measured. One mL of luciferin was added to the leaves, and the materials were kept in the dark for 8 min to quench the fluorescence. A low-light-cooled CCD imaging apparatus (Tanon 5200) was used to capture the LUC image. Primer information is shown in Supplemental Table 6.

### Virus-induced gene silencing

VIGS primers were designed and amplified by PCR based on the full-length coding sequence of the *GhCLE5* gene. The PCR product was connected to a *pTRV2:00* empty carrier. *pTRV2:GhCLE5* was introduced into *A. tumefaciens* GV3101 using a freeze–thaw method. The positive strain and agrobacteria containing the plasmids *pTRV1*, *pTRV2:00,* and *pTRV2:CLA*^52^ were expanded and cultured. After suspension for 3 h, the *pTRV1* was mixed with *pTRV2:00*, *pTRV2:GhCLE5,* and *pTRV2:CLA* at a volume ratio of 1:1. Cotton seedlings with cotyledons that had just flattened were injected with a fungus solution at the back of two thick cotyledons. All cotton seedlings were placed in a light incubator and cultured at 21 to 25 °C for 15 days. The albino phenotype of the silent *pTRV2:CLA* plants was observed 7 to 8 days after injection. The plants lost their green color from the first true leaf, and there was no difference between the injected no-load and noninjected negative controls. The height from the cotyledon to the growing point of the cotton was measured, and Student’s *t* tests were used to determine significant differences between experimental and control plants.

### Promoter activity assay using tobacco leaves

The *GhCLE5* promoter was cloned using *G. hirsutum* acc. TM-1 genomic DNA and ligated into the *pBI121* vector (primer sequences shown in Supplemental Table 6). Using *Agrobacterium tumefaciens* GV3101 as the mediating bacteria, the plasmid was transformed into *Agrobacterium* by heat transformation. The resulting *Agrobacterium* GV3101 single clone was cultivated in liquid LB medium containing kanamycin (50 µg/mL) and rifampin (50 µg/mL) at 28 °C overnight with shaking at 200 rpm. The bacteria were then collected by centrifugation at 4000 rpm/min for 10 min, and resuspended in injection buffer (10 mM MgCl_2_, 10 mM MES, pH 5.7, 150 µM acetosyringone) to a concentration of OD_600_ = 0.8–1. The bacterial injection buffer was incubated in the dark at 28 °C for 3 h and then injected into six-week-old tobacco leaves. After a 72 h cultivation in the dark, the tobacco leaves were harvested for GUS staining. Staining was carried out at 37 °C for 2–3 h, and then the leaves washed with fixative (70% ethanol: glacial acetic acid (V/V) = 9:1). The experiment was repeated three times, with three biological replicates each time.

## Supplementary Information

Supplementary Information
