# Supplementary material for: Functional analysis of the cotton CLE polypeptide signaling gene family in plant growth and development
Source: Sci Rep. 2021 Mar 3;11:5060. doi: 10.1038/s41598-021-84312-8 (PMC7930028; doi:10.1038/s41598-021-84312-8)
Supplement: Supplementary file 1 — Supplementary Information [file 41598_2021_84312_MOESM1_ESM.pdf]

Functional Analysis of the Cotton *CLE* Polypeptide Signaling Gene Family in Plant Growth and Development

Ke Wan[1], Kening Lu[1], Mengtao Gao[1], Ting Zhao[1,2], Yuxin He[1], Dong-Lei Yang[1], Xiaoyuan Tao[2], Guosheng Xiong[1], Xueying Guan[2*]

[1]State Key Laboratory of Crop Genetics and Germplasm Enhancement, Cotton Hybrid R & D Engineering Center (the Ministry of Education), College of Agriculture, Nanjing Agricultural University, Nanjing, Jiangsu, 210095, China.

[2]Zhejiang University, College of Agriculture and Biotechnology, Hangzhou, Zhejiang, 210058, China.

[*]Corresponding author. Correspondence and requests for materials should be addressed to X. Guan (xueyingguan@zju.edu.cn)

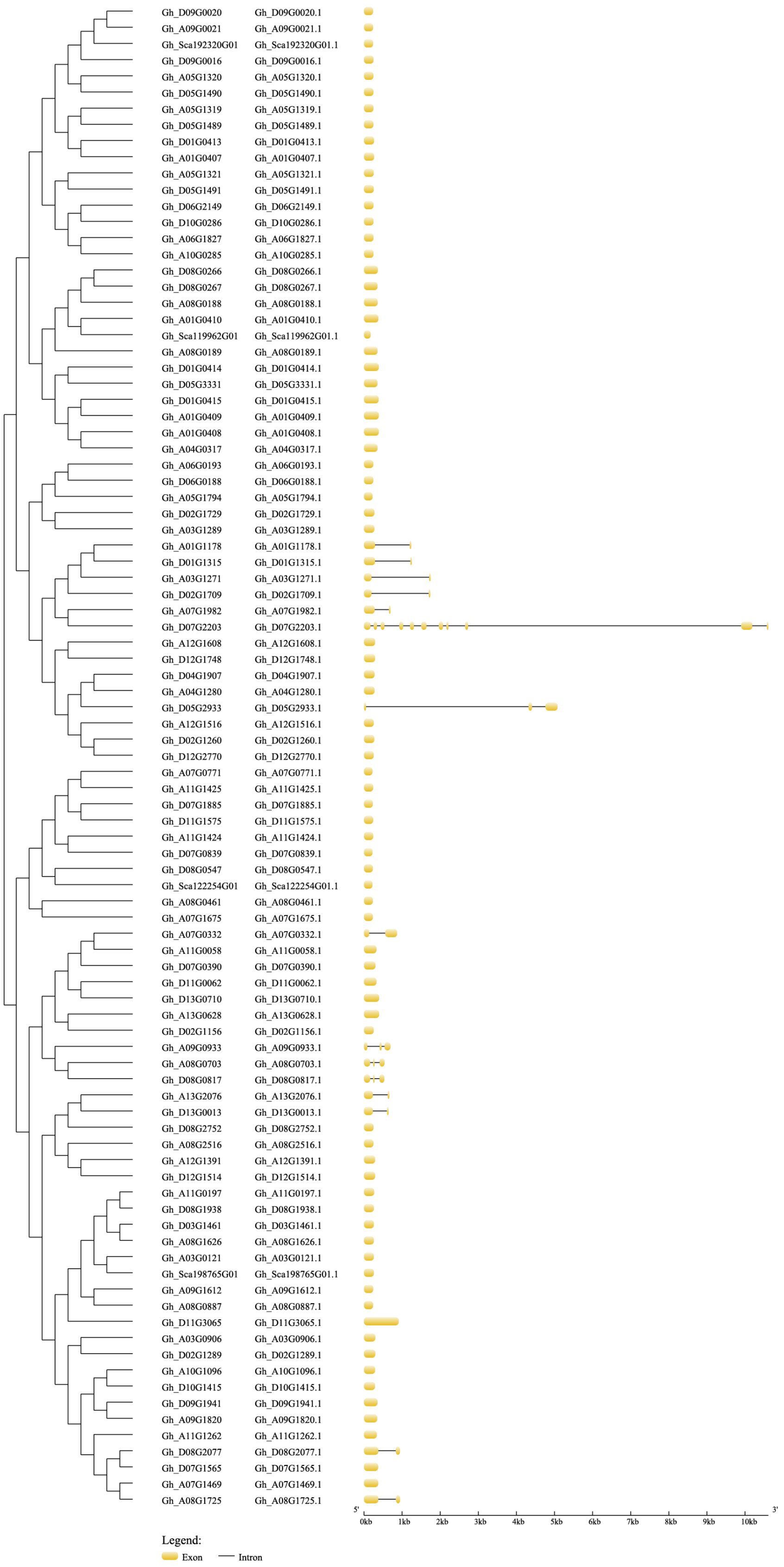

Supplemental Figure 1: Phylogenetic tree of the Upland cotton *CLE* gene family.

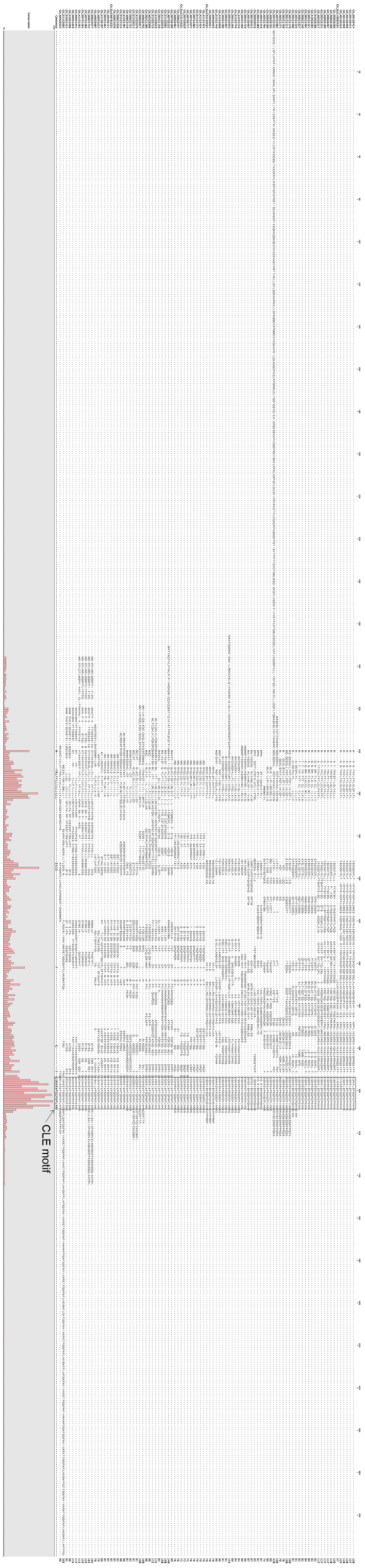

Supplemental Figure 2: The amino acid sequence alignment of Upland cotton *CLE* gene family.

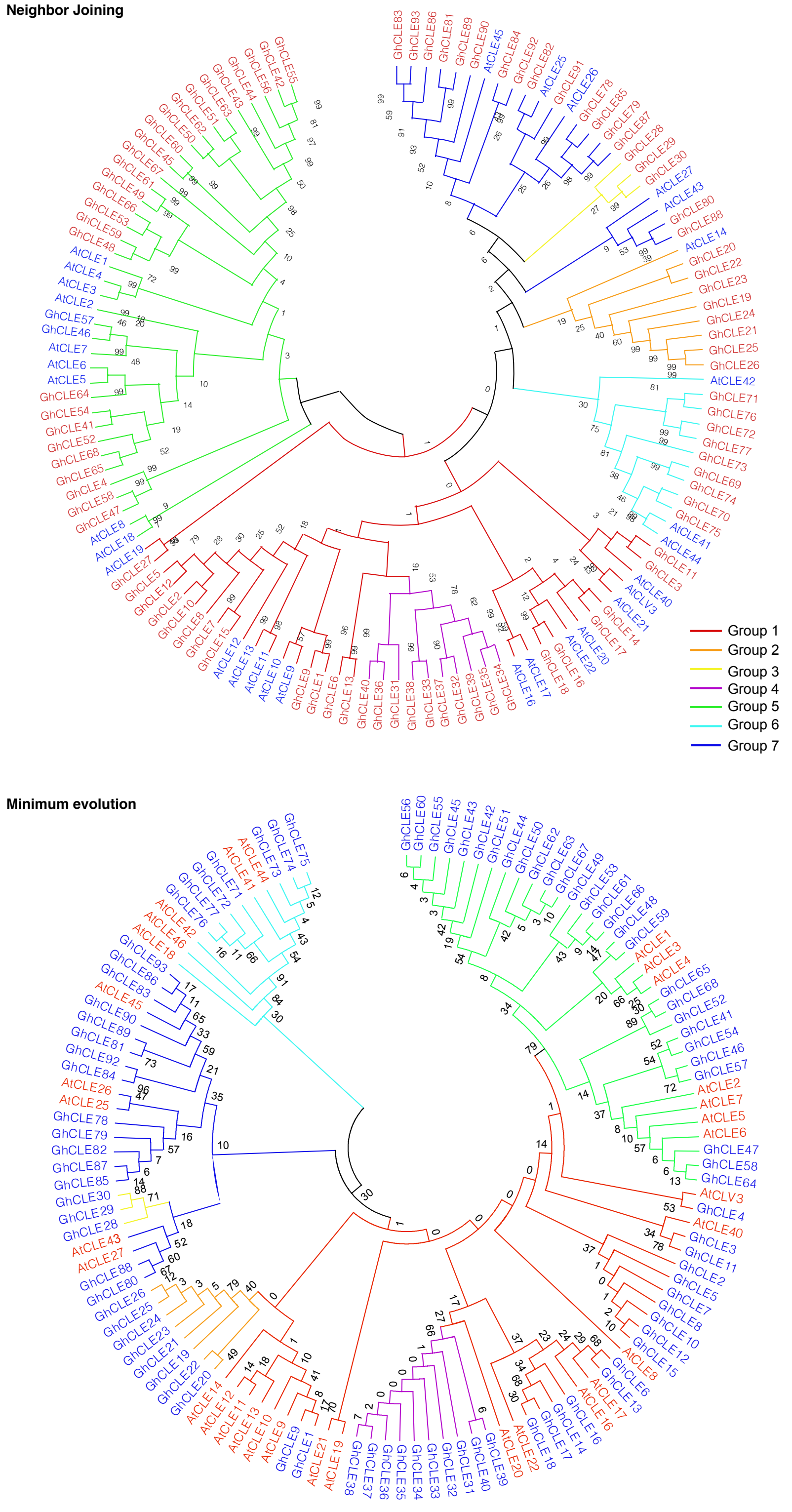

Supplemental Figure 3: Phylogenetic tree of the Upland cotton *CLE* gene family in paralleled with Arabidopsis CLE gene family using the full length of the proteins. The Neighbor Joining and Minimum evolution method were used respectively.

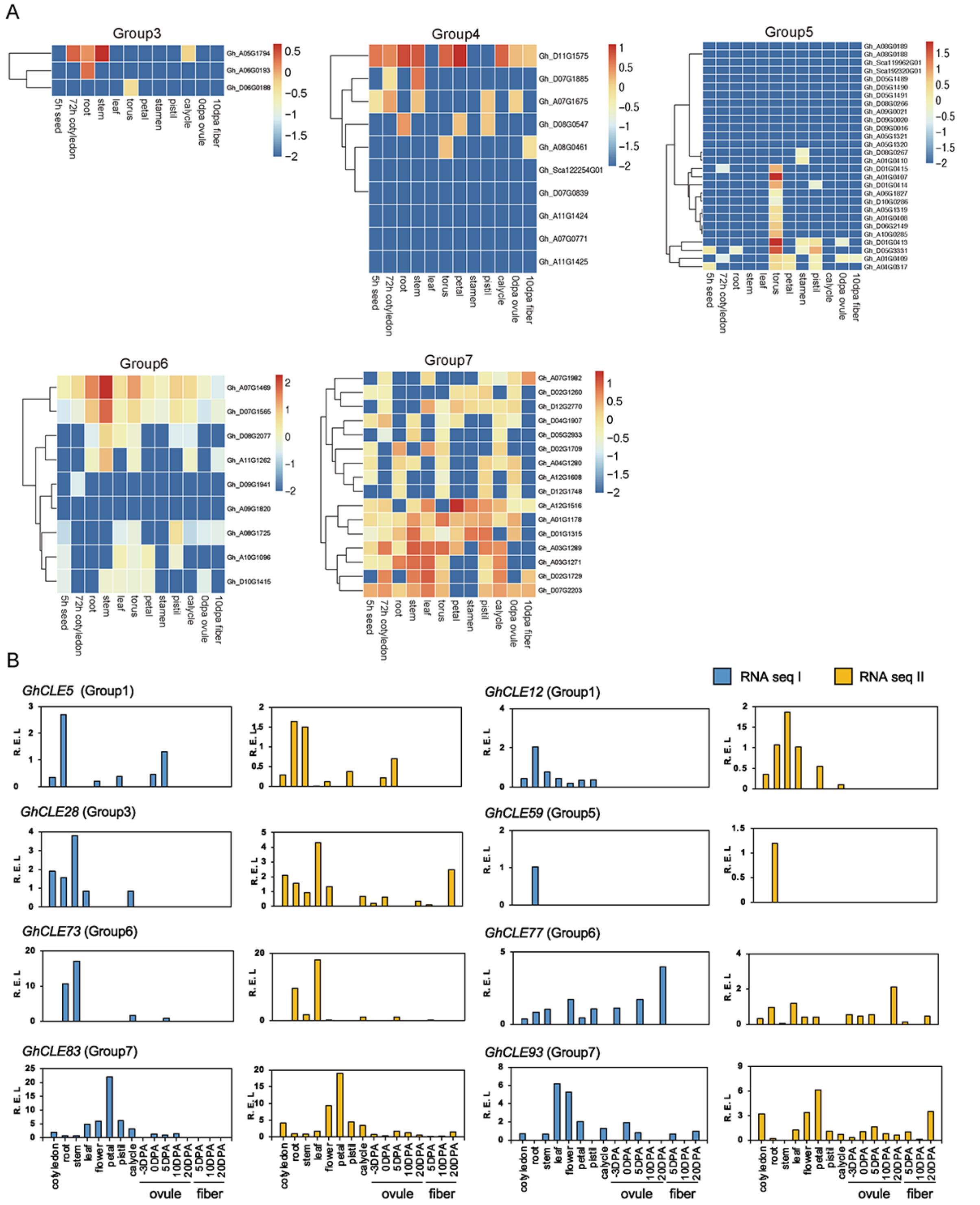

Supplemental Figure 4: The expression pattern of groups of cotton *CLE* gene family. A: The heat maps of cotton *CLE* gene family, Group 2-6. B: The histograms of the expression pattern in cotton tissues for the selected 8 cotton CLE gene members with two RNA seq replicates published in 2015 [44] and 2019 respectively [51].

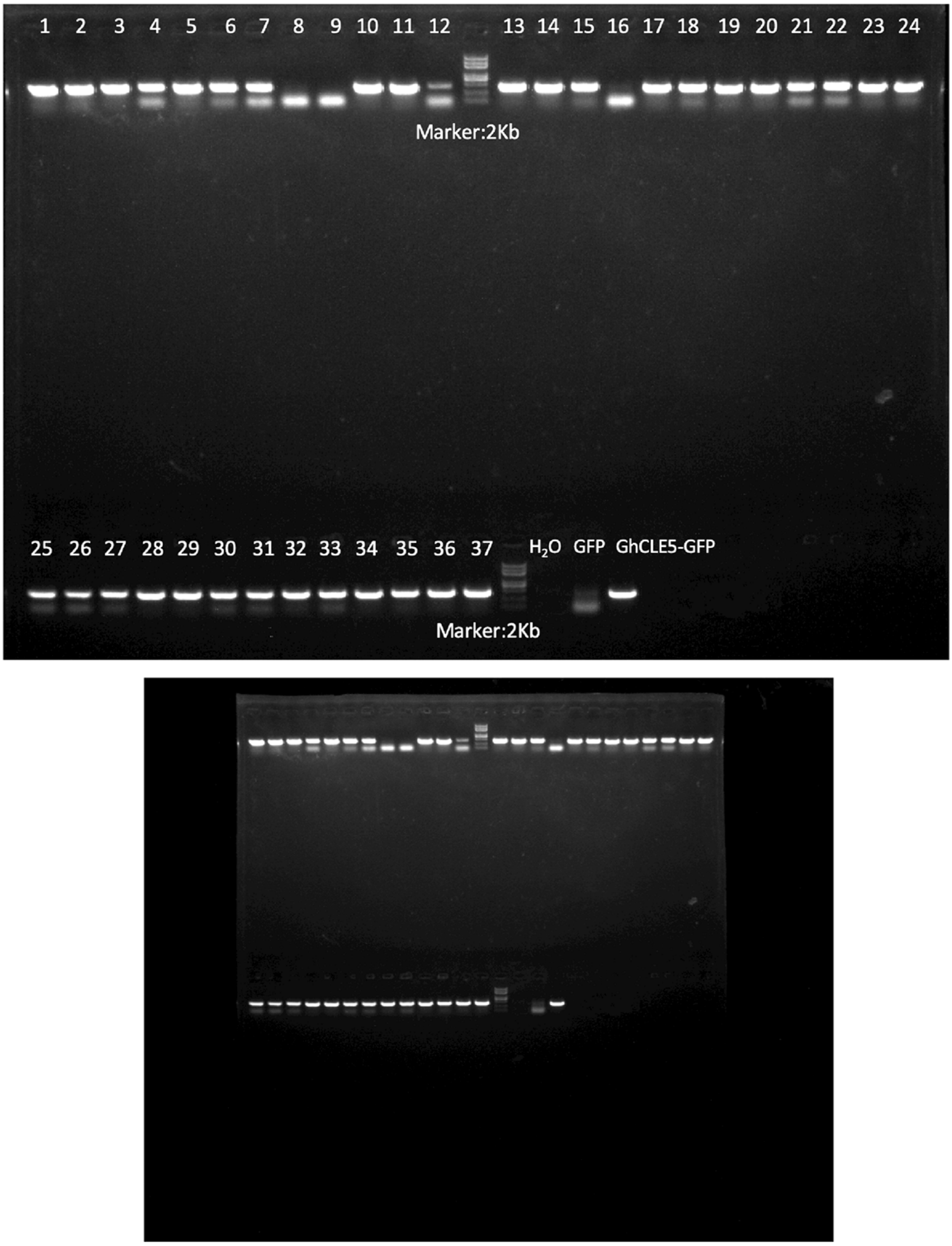

Supplemental Figure 5: The gel image to show the transgenic confirmation of the *GhCLE5* transgenic *Arabidopsis* at T1 generation. A, The gel image show the PCR confirmation tests. The labeled numbers are the transgenic lines of T1 generation. B, The original gel photo of (A) without label and adjusting on brightness, contrast and crop.

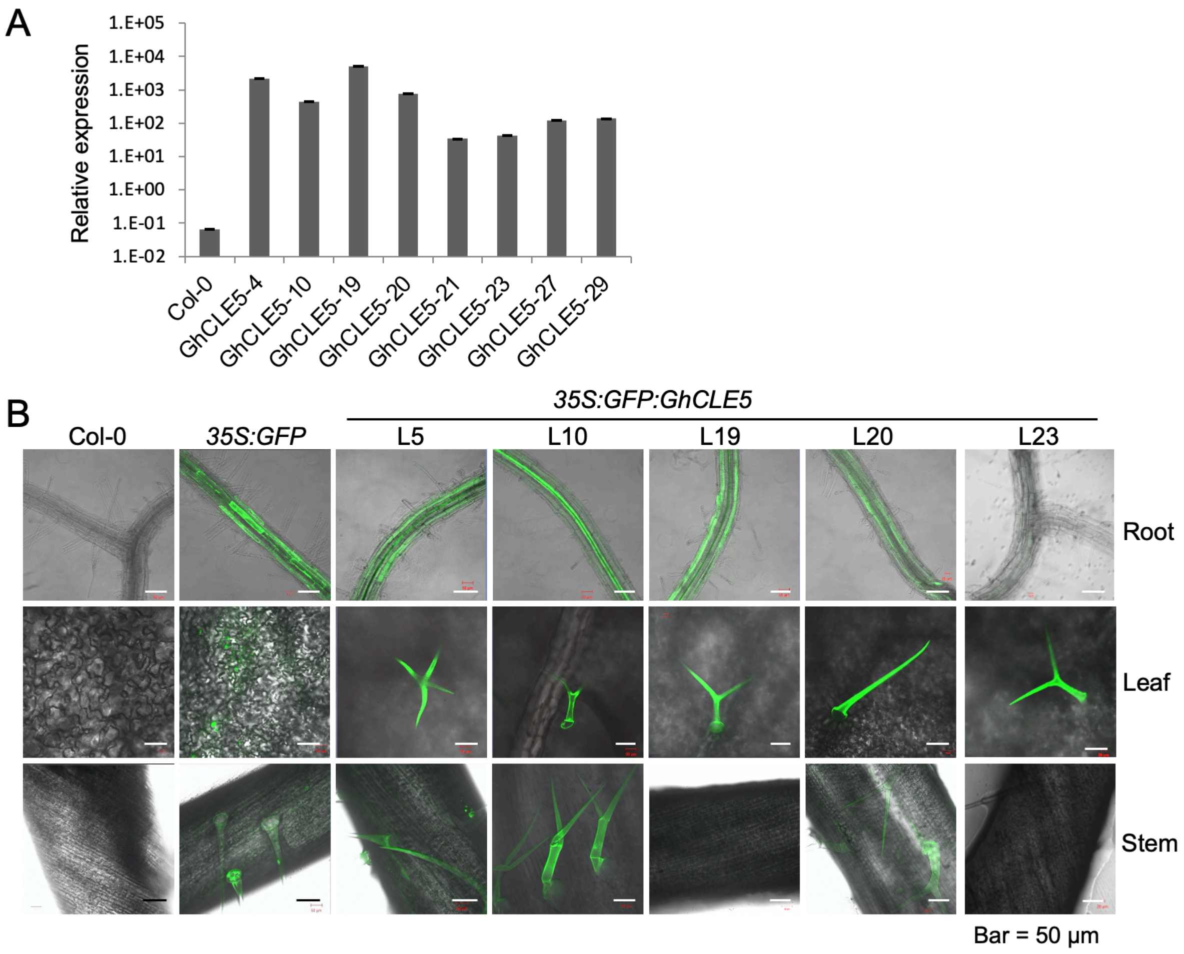

Supplemental Figure 6: The ectopic behavioral pattern of *GhCLE5* in transgenic *Arabidopsis*. A: The relative RNA expression of *GhCLE5* in the transgenic *Arabidopsis* lines. B: The GFP signals represent GhCLE5 protein translated in vascular and trichomes in tissues of root, leaf and stems.

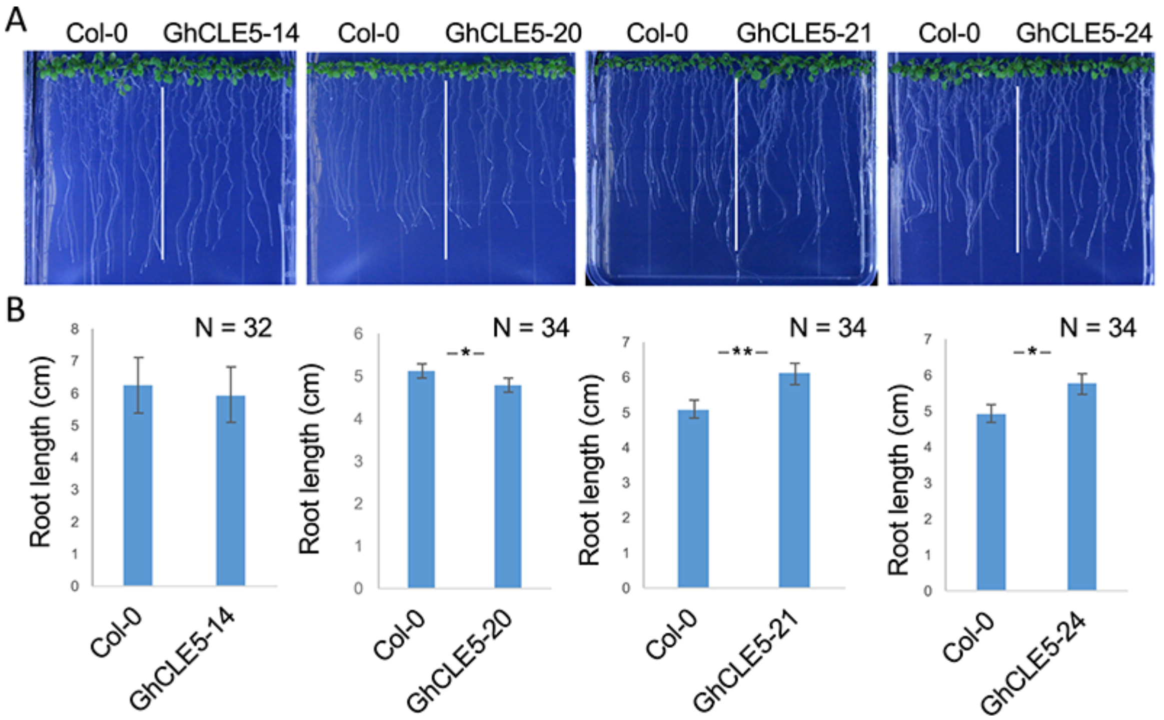

Supplemental Figure 7: The root assay of *35S::GhCLE5::GFP* transgenic *Arabidopsis* lines. A: The images of root growth on 1/2 MS media for 10 days. B: The histograms show the root length of the *35S::GhCLE5::GFP* transgenic Arabidopsis lines in compared with the Col-0 control. *, $p < 0.05$; **, $p < 0.01$, Student's *t*'test. Error bar = Std

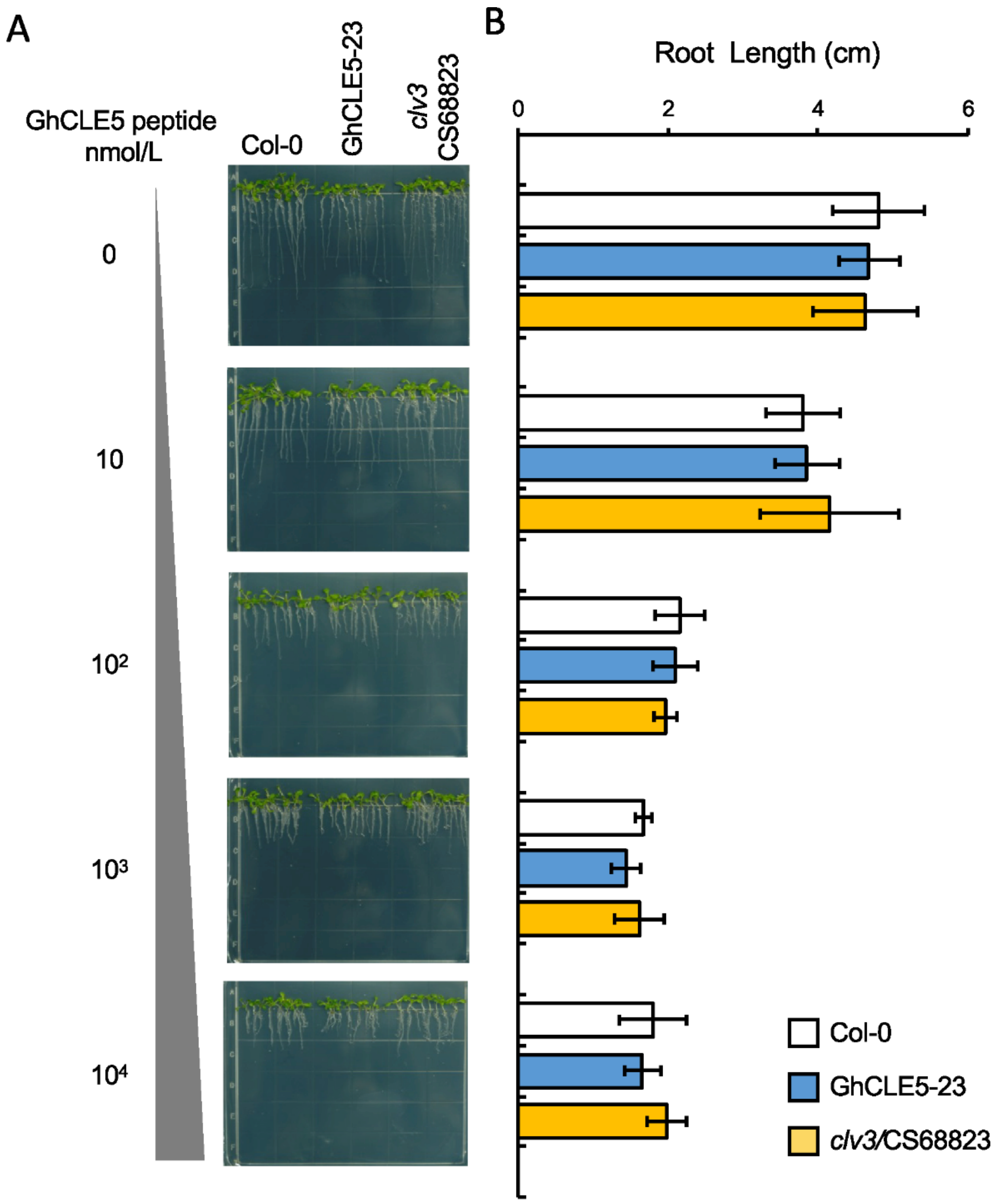

Supplemental Figure 8: The effect of the synthetic peptide of GhCLE5 to the root growth of *Arabidopsis*. A: The photo images of the root growth assay for two weeks on 1/2 MS media with the application of the synthetic GhCLE5 CLE peptide. The concentration of the peptide is indicated on the left. B: The histogram to show the root length of the assay shown in panel (A). N = 50. Error bar = Std

Supplemental table 1: The *CLE* gene family members in upland cotton (Gossypium hirsutum, Texas Marker-1, TM-1) genome

Name	Gene ID (2015)*	Motif	Chromosome	Start	End	Gene ID (2019)**	Chromosome	Start	End	Group
GhCLE1	Gh_A03G0906	KRLVPSGPNPLHN	A03	57302724	57303020	GH_A03G1216	A03	66685220	66684924	Group1
GhCLE2	Gh_A07G0332	KRLVPTGPNPLHH	A07	4277209	4278077	GH_A07G0447	A07	4793086	4793954	Group1
GhCLE3	Gh_A08G0703	KREVPTGPDPLHH	A08	16783067	16783604	GH_A08G0838	A08	17240146	17240683	Group1
GhCLE4	Gh_A09G0933	LRAVPSGPDPLHH	A09	58277166	58277859	GH_A09G1160	A09	64997738	64998222	Group1
GhCLE5	Gh_A11G0058	KRLVPTGPNPLHH	A11	587007	587333	GH_A11G0063	A11	590121	590447	Group1
GhCLE6	Gh_A12G1391	KRIVHTGPNPLHN	A12	72043439	72043729	NA	A12	91153318	91153428	Group1
GhCLE7	Gh_A13G0628	KRLVPTGPNPLHH	A13	15507275	15507670	NA	A13	16309297	16308902	Group1
GhCLE8	Gh_D02G1156	KRLVPTGPNPLHH	D02	34916315	34916569	NA	D02	37604348	37604602	Group1
GhCLE9	Gh_D02G1289	KRLVPSGPNPLHN	D02	42105342	42105638	GH_D02G1405	D02	44631655	44631359	Group1
GhCLE10	Gh_D07G0390	KRLVPTGPNPLHH	D07	4275673	4275972	GH_D07G0449	D07	4559406	4559705	Group1
GhCLE11	Gh_D08G0817	KREVPTGPDPLHH	D08	13073230	13073763	GH_A08G0838	D08	12939458	12938925	Group1
GhCLE12	Gh_D11G0062	KRLVPTGPNPLHH	D11	589759	590085	GH_D11G0068	D11	630871	631197	Group1
GhCLE13	Gh_D12G1514	KRIVHTGPNPLHN	D12	45632507	45632797	NA	D12	47618108	47618398	Group1
GhCLE14	Gh_D13G0013	QRKVYTGPNPLHN	D13	91813	92457	NA	D13	95304	95536	Group1
GhCLE15	Gh_D13G0710	KRLVPTGPNPLHH	D13	10964042	10964437	NA	D13	10706433	10706038	Group1
GhCLE16	Gh_A08G2516	KRKVHTGPNPLHN	scaffold2270_A08	12392	12637	NA	A08	1103950	1104195	Group1
GhCLE17	Gh_A13G2076	QRKVYTGPNPLHN	scaffold3411_A13	127566	128230	NA	A13	126074	126738	Group1
GhCLE18	Gh_D08G2752	KRKVYTGPNPLHN	scaffold4315_D08	113830	114078	NA	D08	1143617	1143865	Group1
GhCLE19	Gh_A03G0121	YRTVPGGPNPLHN	A03	1897260	1897517	GH_A03G0252	A03	3209955	3209698	Group2
GhCLE20	Gh_A08G0887	HLLVPGGPNPLHN	A08	51703132	51703368	NA	A08	74564484	74564720	Group2
GhCLE21	Gh_A08G1626	YRTVPGGPNPLHN	A08	95373267	95373524	GH_D08G2075	A08	60755615	60755358	Group2
GhCLE22	Gh_A09G1612	HKVVPGGPNPLHN	A09	69791982	69792221	GH_A09G1955	A09	76666081	76665842	Group2
GhCLE23	Gh_A11G0197	YRTVPGGPNPLHN	A11	1905655	1905921	NA	A11	1851029	1850763	Group2
GhCLE24	Gh_D03G1461	YRTVPGGPNPLHN	D03	43571648	43571905	GH_A03G0252	D03	50733728	50733985	Group2
GhCLE25	Gh_D08G1938	YRTVPGGPNPLHN	D08	57539690	57539947	GH_D08G2075	D08	60755615	60755358	Group2
GhCLE26	Gh_Sca198765G01	YRTVPGGPNPLHN	scaffold198765	4	261	GH_D08G2075	D08	60755615	60755358	Group2
GhCLE27	Gh_D11G3065	KRETPGGPNPIHN	D11	62564959	62565867	GH_D11G3471	D11	67819487	67820395	Group2
GhCLE28	Gh_A05G1794	KREVPNASDPLHN	A05	18884525	18884749	GH_A05G2078	A05	19945387	19945163	Group3
GhCLE29	Gh_A06G0193	KRKVPNAADPLHN	A06	2167534	2167776	GH_A06G0222	A06	2283207	2282965	Group3
GhCLE30	Gh_D06G0188	KRKVPNAADPLHN	D06	1873946	1874188	GH_D06G0206	D06	2033601	2033359	Group3
GhCLE31	Gh_A07G0771	KRRVPTGSNPLHN	A07	12705812	12706033	GH_A07G0933	A07	10717469	10717248	Group4
GhCLE32	Gh_A07G1675	KRRVPTGSNPLHN	A07	67994221	67994451	NA	A07	85095556	85095786	Group4
GhCLE33	Gh_A08G0461	KRRVPTGSNPLHN	A08	6350090	6350320	GH_A08G0564	A08	6698817	6698587	Group4
GhCLE34	Gh_A11G1424	KRRVPTGSNPLHN	A11	19044407	19044646	GH_A11G1609	A11	18870359	18870120	Group4
GhCLE35	Gh_A11G1425	KRRVPTGSNPLHN	A11	19046318	19046557	GH_A11G1609	A11	18870359	18870120	Group4
GhCLE36	Gh_D07G0839	KRRVPTGSNPLHN	D07	10733960	10734181	GH_D07G0936	D07	10717469	10717248	Group4
GhCLE37	Gh_D07G1885	KRRVPTGSNPLHN	D07	46224446	46224676	NA	D07	49732587	49732817	Group4
GhCLE38	Gh_D08G0547	KRRVPTGSNPLHN	D08	6336823	6337053	GH_D08G0564	D08	6101016	6100786	Group4
GhCLE39	Gh_D11G1575	KRRVPTGSNPLHN	D11	16177374	16177613	NA	D11	85095556	85095786	Group4
GhCLE40	Gh_Sca122254G01	KRRVPTGSNPLHN	scaffold122254	37	258	GH_D07G0936	D07	10717469	10717248	Group4
						GH_A07G0933	A07	13129314	13129093	
GhCLE41	Gh_A01G0407	ERISPGGPDPQHH	A01	6428720	6428983	GH_A01G0537	A01	575321	575073	Group5
GhCLE42	Gh_A01G0408	KRLSPGGPDPHHH	A01	6436261	6436647	GH_A01G0538	A01	6569847	6569461	Group5
GhCLE43	Gh_A01G0409	KRLSPGGPDPHHH	A01	6455864	6456250	GH_A01G0539	A01	6589362	6588976	Group5
GhCLE44	Gh_A01G0410	KRRSPGGPDPHHH	A01	6474462	6474839	GH_A01G0542	A01	6607056	6606679	Group5
GhCLE45	Gh_A04G0317	KRLSPGGPDPHHH	A04	7620656	7621009	GH_A04G0408	A04	8134989	8135342	Group5
GhCLE46	Gh_A05G1319	SRISPGGPDPQHD	A05	13515175	13515420	GH_A05G1574	A05	14719745	14719500	Group5
GhCLE47	Gh_A05G1320	KRVSPGGPDPQHH	A05	13534683	13534928	NA	A05	14739470	14739225	Group5
GhCLE48	Gh_A05G1321	KRLSPGGPDPKHH	A05	13543377	13543631	GH_A05G1575	A05	14747339	14747085	Group5
GhCLE49	Gh_A06G1827	KRRSPGGPDPKHH	A06	103154239	103154487	GH_A06G2282	A06	125647909	125647661	Group5
GhCLE50	Gh_A08G0188	KRRSPGGPDPHHH	A08	1884282	1884638	GH_A08G0262	A08	2454463	2454819	Group5
GhCLE51	Gh_A08G0189	KRQSPGGPDPHHH	A08	1887614	1887967	GH_A08G0263	A08	2457795	2458148	Group5
GhCLE52	Gh_A09G0021	DRVSPGGPDHEHH	A09	564471	564707	GH_A09G0026	A09	710623	710387	Group5
GhCLE53	Gh_A10G0285	KRRSPGGPDPKHH	A10	2579248	2579496	GH_A10G0324	A10	2642834	2643082	Group5
GhCLE54	Gh_D01G0413	ERISPGGPDPQHH	D01	4857948	4858211	GH_D01G0519	D01	5711512	5711249	Group5
GhCLE55	Gh_D01G0414	KRLSPGGPDPHHH	D01	4863769	4864155	GH_D01G0520	D01	5717607	5717221	Group5
GhCLE56	Gh_D01G0415	KRLSPGGPDPHHH	D01	4877939	4878322	GH_D01G0521	D01	5731791	5731408	Group5
GhCLE57	Gh_D05G1489	SRISPGGPDPQHD	D05	13351848	13352093	GH_D05G1604	D05	13546491	13546246	Group5
GhCLE58	Gh_D05G1490	KRVSPGGPDPQHH	D05	13404080	13404325	NA	D05	13574400	13574155	Group5
GhCLE59	Gh_D05G1491	KRLSPGGPDPKHH	D05	13424211	13424465	GH_D05G1605	D05	13595632	13595378	Group5
GhCLE60	Gh_D05G3331	KRLSPGGPDPHHH	D05	53487599	53487952	GH_D05G3656	D05	55714754	55714401	Group5
GhCLE61	Gh_D06G2149	KRRSPGGPDPKHH	D06	63323457	63323705	GH_D06G2321	D06	64526055	64525807	Group5
GhCLE62	Gh_D08G0266	KRRSPGGPDPHHH	D08	2551681	2552043	GH_D08G0278	D08	2445579	2445941	Group5
GhCLE63	Gh_D08G0267	KRRSPGGPDPHHH	D08	2555462	2555815	GH_D08G0279	D08	2449360	2449713	Group5
GhCLE64	Gh_D09G0016	DRVSPGGPDPQHH	D09	572937	573185	GH_D09G0021	D09	575321	575073	Group5
GhCLE65	Gh_D09G0020	DRVSPGGPDHEHH	D09	667869	668105	GH_D09G0025	D09	670590	670354	Group5
GhCLE66	Gh_D10G0286	KRRSPGGPDPKHH	D10	2446306	2446554	GH_D10G0337	D10	2646967	2647215	Group5
GhCLE67	Gh_Sca119962G01	KRRSPGGPDPHHH	scaffold119962	202	375	GH_D01G0536	D01	6000511	6000338	Group5
GhCLE68	Gh_Sca192320G01	DRVSPGGPDHEHH	scaffold192320	24	260	GH_D09G0025	D09	670590	670354	Group5
						GH_A09G0026	A09	710623	710387	
GhCLE69	Gh_A07G1469	AHEVPSGPNPISN	A07	43066097	43066468	GH_A07G1703	A07	43634747	43635118	Group6
GhCLE70	Gh_A08G1725	AHEVPSGPNPISN	A08	97224588	97225535	GH_D08G2225	A08	118352563	118352943	Group6
GhCLE71	Gh_A09G1820	AHEVPSGPNPESN	A09	71553498	71553845	GH_A09G2204	A09	78960017	78960364	Group6
GhCLE72	Gh_A10G1096	VHEVPSGPNPESN	A10	51520862	51521152	GH_A10G1331	A10	65729884	65730174	Group6
GhCLE73	Gh_A11G1262	AHEVPSGPNPISN	A11	15689774	15690109	GH_A11G1427	A11	15528143	15528478	Group6
GhCLE74	Gh_D07G1565	AHEVPSGPNPISN	D07	29430991	29431362	GH_A07G1703	D07	30074521	30074892	Group6
GhCLE75	Gh_D08G2077	AHEVPSGPNPISN	D08	59612034	59612977	GH_D08G2225	D08	62628471	62628848	Group6
GhCLE76	Gh_D09G1941	AHEVPSGPNPESN	D09	46876891	46877244	GH_D09G2138	D09	48199779	48200132	Group6
GhCLE77	Gh_D10G1415	VHEVPSGPNPESN	D10	29736548	29736835	GH_D10G1555	D10	28775091	28774804	Group6
GhCLE78	Gh_A01G1178	KRRVPNGPDPIHN	A01	58937135	58938373	GH_A01G1502	A01	69915153	69916391	Group7
GhCLE79	Gh_A03G1271	KRRVPNGPDPIHN	A03	89043090	89044838	NA	A03	100084058	100082552	Group7
GhCLE80	Gh_A03G1289	KRRVPSCPDPLHN	A03	89715101	89715373	GH_A03G1683	A03	100749894	100749622	Group7
GhCLE81	Gh_A04G1280	KRRFRRGPDPIHN	A04	62850194	62850469	GH_A04G1768	A04	87633494	87633769	Group7
GhCLE82	Gh_A07G1982	KRRVPNGPDPIHN	A07	75953942	75954637	GH_A07G2451	A07	94356866	94356171	Group7
GhCLE83	Gh_A12G1516	KRRVRRGSDPIHN	A12	74723276	74723530	GH_A12G1846	A12	93944017	93944271	Group7
GhCLE84	Gh_A12G1608	KRSVKKGSDPIHN	A12	76988145	76988435	GH_D12G1958	A12	96618041	96617751	Group7
GhCLE85	Gh_D01G1315	KRRVPNGPDPIHN	D01	37087307	37088562	GH_D01G1590	D01	40665687	40664432	Group7
GhCLE86	Gh_D02G1260	KRRVRRGSDPIHN	D02	41282754	41283023	GH_D02G1373	D02	43826463	43826194	Group7
GhCLE87	Gh_D02G1709	RRRVPNGPDPIHN	D02	58515338	58517077	NA	D02	60665001	60663277	Group7
GhCLE88	Gh_D02G1729	KRRVPSCPDPLHN	D02	59132343	59132615	GH_D02G1846	D02	61232326	61232054	Group7
GhCLE89	Gh_D04G1907	KRRFRRGPDPIHN	D04	51391312	51391587	GH_D04G2115	D04	56868117	56868392	Group7
GhCLE90	Gh_D05G2933	KRRVRNGPDPIHN	D05	34832114	34837192	GH_D05G3134	D05	34084340	34079338	Group7
GhCLE91	Gh_D07G2203	KRRVPNGPDPIHN	D07	52848785	52859398	GH_D07G2398	D07	56081479	56064678	Group7
GhCLE92	Gh_D12G1748	KRSVKKGSDPIHN	D12	49785229	49785519	GH_D12G1958	D12	51609915	51609625	Group7
GhCLE93	Gh_D12G2770	KRRVRRGSDPIHN	scaffold4593_D12	6316	6570	GH_D12G1842	D12	49680177	49680431	Group7

*, Tianzhen, Z. et al. Sequencing of allotetraploid cotton (Gossypium hirsutum L. acc. TM-1) provides a resource for fiber improvement. Nature Biotechnology 33, 531-537 (2015).
**, Hu, Y. et al. Gossypium barbadense and Gossypium hirsutum genomes provide insights into the origin and evolution of allotetraploid cotton. Nature genetics 51, 739-748, doi:10.1038/s41588-019-0371-5 (2019).

Supplemental Table 2: The Ka/Ks value of GhCLE homeologous pairs.

Pair	Ka	Ks	Ka/Ks
Gh_A01G0407-Gh_D01G0413	NA	0.0535942	NA
Gh_A01G0408-Gh_D01G0414	0.016977	0.0479374	0.35414937
Gh_A03G0121-Gh_D03G1461	0.0151285	0.0974913	0.155177949
Gh_A03G0906-Gh_D02G1289	0.0138331	0.0271074	0.510307149
Gh_A03G1271-Gh_D02G1709	0.028973	0.0535268	0.541280256
Gh_A03G1289-Gh_D02G1729	0.0282592	0.0188731	1.497326883
Gh_A04G0317-Gh_D05G3331	0.0472431	0.0757686	0.623518186
Gh_A04G1280-Gh_D04G1907	0.0142195	0.0168503	0.843872216
Gh_A05G1319-Gh_D05G1489	0.0163153	0.0177158	0.920946274
Gh_A05G1320-Gh_D05G1490	0.0194499	0.0423551	0.459210343
Gh_A05G1321-Gh_D05G1491	0.0210071	0.0171704	1.223448493
Gh_A05G1794-Gh_D05G1989	0.03497	0.0676237	0.517126392
Gh_A06G0193-Gh_D06G0188	0.00547159	0.0178888	0.305866799
Gh_A06G1827-Gh_D06G2149	0.0267598	0.0557887	0.479663444
Gh_A07G0332-Gh_D07G0390	0.00867545	0.047538	0.182495057
Gh_A07G0771-Gh_D07G0839	NA	NA	NA
Gh_A07G1469-Gh_D07G1565	0.0107926	0.0463317	0.232942025
Gh_A07G1675-Gh_D07G1885	0.0416443	0.0371363	1.121390661
Gh_A07G1982-Gh_D07G2203	0.00411569	0.0273903	0.150260859
Gh_A08G0188-Gh_D08G0266	0.0150001	0.0488019	0.307367131
Gh_A08G0461-Gh_D08G0547	0.0207312	0.0642654	0.322587271
Gh_A08G0703-Gh_D08G0817	0.00805966	0.013013	0.619354492
Gh_A08G1626-Gh_D08G1938	NA	NA	NA
Gh_A08G1725-Gh_D08G2077	NA	0.0087168	NA
Gh_A09G0021-Gh_D09G0020	NA	NA	NA
Gh_A09G1820-Gh_D09G1941	0.0160641	0.103231	0.155613139
Gh_A10G0285-Gh_D10G0286	0.0160976	0.0175561	0.916923463
Gh_A10G1096-Gh_D10G1415	0.0290141	0.070629	0.410795849
Gh_A11G0058-Gh_D11G0062	0.00406952	0.0673668	0.060408391
Gh_A11G1262-Gh_D11G1411	0.0240318	0.0256512	0.936868451
Gh_A11G1424-Gh_D11G1575	0.00570223	NA	NA
Gh_A11G2709-Gh_D11G3065	0.0693706	0.144184	0.481125506
Gh_A12G1391-Gh_D12G1514	0.0181774	0.0474327	0.383225075
Gh_A12G1608-Gh_D12G1748	0.0233262	NA	NA
Gh_D13G0013-Gh_A13G2076	NA	0.0321867	NA

Supplemetnal Table 3: The comparison of the the CLE gene members in allotetraploid cotton (*Gossypium hirsutm* , Gh) and the D diploid cotton (*Gossypium raimondii* , Gr).

Number code	ID in Gosypium ramondii (Gr)	CLE reported in Gr*	pep length	Gene ID in Gossypium hirsutum (Gh.v1)	pep length	Similarity %	CLE in Gh D subgenome
1	Gorai.002G063600.1	Gorai.002G063600.1	87	Gh_D01G0413	87	98.85	Gh_D01G0413
2	Gorai.002G165000.1	Gorai.002G165000.1	109	Gh_D01G1315	109	99.08	Gh_D01G1315
3	Gorai.005G141500.1	Gorai.005G141500.1	89	Gh_D02G1260	89	98.88	Gh_D02G1260
4	Gorai.005G144400.1	Gorai.005G144400.1	98	Gh_D02G1289	98	98.98	Gh_D02G1289
5	Gorai.005G188200.1	Gorai.005G188200.1	78	Gh_D02G1709	78	98.72	Gh_D02G1709
6	Gorai.005G190400.1	Gorai.005G190400.1	90	Gh_D02G1729	90	97.78	Gh_D02G1729
7	Gorai.003G160300.1	Gorai.003G160300.1	85	Gh_D03G1461	85	100	Gh_D03G1461
8	Gorai.012G187200.1	Gorai.012G187200.1	91	Gh_D04G1907	91	98.9	Gh_D04G1907
9	Gorai.009G324600.1	Gorai.009G324600.1	135	Gh_D05G2933	153	98.52	Gh_D05G2933
10	Gorai.009G372900.1	Gorai.009G372900.1	117	Gh_D05G3331	117	96.58	Gh_D05G3331
11	Gorai.010G023900.1	Gorai.010G023900.1	80	Gh_D06G0188	80	97.5	Gh_D06G0188
12	Gorai.010G242300.1	Gorai.010G242300.1	82	Gh_D06G2149	82	96.34	Gh_D06G2149
13	Gorai.001G045900.1	Gorai.001G045900.1	115	Gh_D07G0390	99	98.99	Gh_D07G0390
14	Gorai.001G095900.1	Gorai.001G095900.1	73	Gh_D07G0839<br>Gh_Sca122254G01	73	98.63	Gh_D07G0839<br>Gh_Sca122254G01
15	Gorai.001G186300.1	Gorai.001G186300.1	123	Gh_D07G1565	123	100	Gh_D07G1565
16	Gorai.001G215800.1	Gorai.001G215800.1	76	Gh_D07G1885	76	100	Gh_D07G1885
17	Gorai.001G257800.1	Gorai.001G257800.1	106	Gh_D07G2203	429	99.06	Gh_D07G2203
18	Gorai.004G030000.1	Gorai.004G030000.1	120	Gh_D08G0266	120	97.5	Gh_D08G0266
19	Gorai.004G062300.1	Gorai.004G062300.1	76	Gh_D08G0547	76	100	Gh_D08G0547
20	Gorai.004G092000.1	Gorai.004G092000.1	109	Gh_D08G0817	109	98.17	Gh_D08G0817
21	Gorai.004G209500.1	Gorai.004G209500.1	85	Gh_D08G1938<br>Gh_Sca198765G01	85	98.82	Gh_D08G1938<br>Gh_Sca198765G01
22	Gorai.004G225300.1	Gorai.004G225300.1	161	Gh_D08G2077	161	98.76	Gh_D08G2077
23	Gorai.004G015200.1	Gorai.004G015200.1	82	Gh_D08G2752	82	98.78	Gh_D08G2752
24	Gorai.006G002100.1	Gorai.006G002100.1	82	Gh_D09G0016	82	100	Gh_D09G0016
25	Gorai.006G002700.1	Gorai.006G002700.1	78	Gh_D09G0020<br>Gh_Sca192320G01	78	100	Gh_D09G0020<br>Gh_Sca192320G01
26	Gorai.006G221500.1	Gorai.006G221500.1	118	Gh_D09G1941	117	96.61	Gh_D09G1941
27	Gorai.011G159800.1	Gorai.011G159800.1	105	Gh_D10G1415	95	94.68	Gh_D10G1415
28	Gorai.007G007600.1	Gorai.007G007600.1	108	Gh_D11G0062	108	98.15	Gh_D11G0062
29	Gorai.007G171200.1	Gorai.007G171200.1	79	Gh_D11G1575	79	100	Gh_D11G1575
30	Gorai.007G349100.1	Gorai.007G349100.1	453	Gh_D11G3065	302	90.45	Gh_D11G3065
31	Gorai.008G167000.1	Gorai.008G167000.1	96	Gh_D12G1514	96	98.96	Gh_D12G1514
32	Gorai.008G193000.1	Gorai.008G193000.1	96	Gh_D12G1748	96	98.96	Gh_D12G1748
33	Gorai.008G181100.1	Gorai.008G181100.1	84	Gh_D12G2770	84	100	Gh_D12G2770
34	Gorai.013G001300.1	Gorai.013G001300.1	91	Gh_D13G0013	91	100	Gh_D13G0013
35	Gorai.013G079400.1	Gorai.013G079400.1	131	Gh_D13G0710	131	96.95	Gh_D13G0710
36	Gorai.009G164400.1	Gorai.009G164400.1	81	Gh_D05G1490	81	97.53	Gh_D05G1490
37	Gorai.009G164500.1	Gorai.009G164500.1	84	Gh_D05G1491	84	100	Gh_D05G1491
38	Gorai.002G064000.1	Gorai.002G064000.1	125	Gh_Sca119962G01	57	100	Gh_Sca119962G01
39	Gorai.011G033200.1	Gorai.011G033200.1	82	Gh_D10G0286	82	98.78	Gh_D10G0286
40	Gorai.004G030100.1	Gorai.004G030100.1	117	Gh_D08G0267	117	94.87	Gh_D08G0267
41	Gorai.002G063800.1	Gorai.002G063800.1	127	Gh_D01G0415	127	99.21	Gh_D01G0415
42	Gorai.002G063700.1	Gorai.002G063700.1	128	Gh_D01G0414	128	100	Gh_D01G0414
1	Gorai.010G202200.1	Gorai.010G202200.1	85	Gh_D06G1815	83	91.76	NA
2	Gorai.009G216600.1	Gorai.009G216600.1	74	Gh_D05G1989	74	94.59	NA
3	Gorai.008G278200.1	Gorai.008G278200.1	118	Gh_D12G2471	177	98.31	NA
4	Gorai.007G296300.1	Gorai.007G296300.1	74	Gh_D11G2714	74	95.95	NA
5	Gorai.007G153500.1	Gorai.007G153500.1	111	Gh_D11G1411	111	97.3	NA
6	Gorai.007G023000.1	Gorai.007G023000.1	88	Gh_D11G0208	88	98.86	NA
7	Gorai.007G022900.1	Gorai.007G022900.1	78	Gh_D11G0207	79	100	NA
8	Gorai.004G119100.1	Gorai.004G119100.1	83	Gh_D08G1080	83	98.8	NA
9	Gorai.013G091800.1	Gorai.013G091800.1	80	Gh_D13G0832	129	99.18	NA
10	Gorai.009G360400.1	Gorai.009G360400.1	93	Gh_D05G3243	103	73.79	NA
1	Gorai.006G197600.1	Gorai.006G197600.1	79	Gh_A09G1612	79	96.2	NA
2	Gorai.006G114900.1	Gorai.006G114900.1	98	Gh_A09G0933	98	98.98	NA
3	Gorai.005G160900.1	Gorai.005G160900.1	170	Gh_A03G1045	170	96.47	NA
1	Chr09:12585031-12584786	NA	DNA<br>length：246	Gh_D05G1489	81	99.59	Gh_D05G1489
2	Chr05:35599465-35599211	NA	DNA<br>length：255	Gh_D02G1156	84	99.22	Gh_D02G1156

*: Goad, D. M., C. Zhu, E. A. Kellogg. Comprehensive identification and clustering of CLV3/ESR-related (CLE) genes in plants finds groups with potentially shared function. New Phytol, 2017, 216(2): 605-616

Supplemental table 4: The selection of GhCLE genes in cellular localization assays in plant

Group	Gene ID	Gene Name	Homolog in Arabidopsis	Similarity with Arabidopsis homologs	Localization	
					Tabacco	*Arabidopsis*
Group1	Gh_A11G0058	GhCLE5	CLE12	69.8%	Cell membrane	leaf epidermal cell, trichome
	Gh_D11G0062	GhCLE12	NA	NA	Cell membrane	Root, leaf, anther, stigma, trichome
Group2	Gh_A08G0887	GhCLE20	CLE10	41.0%	Cell membrane	mesophyll cell, leaf epidermal cell, trichome
	Gh_A11G0197	GhCLE23	NA	NA	Cell membrane, nuclei	
	Gh_D11G3065	GhCLE27	NA	NA	NA	
Group3	Gh_A05G1794	GhCLE28	NA	NA	NA	
Group4	Gh_A07G1675	GhCLE32	NA	NA	Cell membrane	trichome
	Gh_D11G1575	GhCLE39	NA	NA	Cell membrane	
Group5	Gh_D05G1490	GhCLE58	NA	NA	Cell membrane, nuclei	
	Gh_D05G1491	GhCLE59	NA	NA	Cell membrane	
	Gh_D09G0020	GhCLE65	NA	NA	Cell membrane, nuclei	
Group6	Gh_A11G1262	GhCLE73	CLE44	62.6%	Cell membrane	mesophyll cell
	Gh_D10G1415	GhCLE77	NA	NA	Cell membrane, nuclei	
Group7	Gh_A03G1271	GhCLE79	NA	NA	Cell membrane	vein, root, root tip, mesophyll cell, trichome
	Gh_A12G1516	GhCLE83	CLE25	50.0%	Cell membrane	mesophyll cell, trichome, root,
	Gh_D12G2770	GhCLE93	CLE45	53.6%	Cell membrane	trichome

Supplemental table 5: The phenotypes shown in the *35S::GhCLE5* transgenic plants in Arabidopsis thaliana in T1 generation

Transgenic line	Descriptioni of phenotype
GhCLE5-3	*clv3* -like florensense
GhCLE5-4	dwarf, *clv3* -like florensense
GhCLE5-5	dwarf and twisted stem, *clv3* -like florensense, club silique
GhCLE5-10	fasciated stem, *clv3* -like florensense
GhCLE5-19	regenerated rossette leaf on cauline leaf
GhCLE5-20	fasciated stem
GhCLE5-21	*clv3* -like florensense, club silique
GhCLE5-23	*clv3* -like floresense, club silique
GhCLE5-24	fasciated stem, *clv3* -like floresense, club silique
GhCLE5-27	fasciated stem, club silique
GhCLE5-29	fasciated stem
GhCLE5-37	fasciated stem

Supplemental Table 6: The primers used for cloning genes and constructs

**Gene ID**	**F primer(5'to 3')**	**R primer(5'to 3')**	**function in experiment**
Gh-D11G0062(*GhCLE12*)	ATGGCCATCAAAGTTAGCAC	TCAATGGTGCAATGGGTTTG	TA clone
Gh_A11G0058(*GhCLE5*)	ATGGCCATCAAAGTTAGCACC	TCAATGGTGCAATGGGTTTGG	TA clone
Gh_A11G0197(*GhCLE23*)	ATGATCATCATTTGTAACCCCA	TCAGTTGTGGAGGGGGTTTGG	TA clone
Gh_A08G0887(*GhCLE20*)	ATGTCCAGTTTCCGTGATTCC	TCAGTTGTGCAGTGGGTTTTC	TA clone
Gh_D11G3065(*GhCLE27*)	ATGGAGTCGTCGTTGATTTTC	TCATATGCCCGGAGGCGGATA	TA clone
Gh_A07G1675(*GhCLE32*)	ATGAATCGCTTTCAGCTTTG	TCATCGCCTGTTGTGTAGTGG	TA clone
Gh_D11G1575(*GhCLE39*)	ATGGATCGTTTTCAGCTTTGC	TCATCGCTTGTTGTGCAAGGG	TA clone
Gh_D05G1490(*GhCLE58*)	ATGGCAATTTCGAAATTCCAT	CTAATGATGGATGGAGTGATG	TA clone
Gh_D05G1491(*GhCLE59*)	ATGGCTCAATGGCGATTTCTT	TCAGTGATGTTTGGGATCAGG	TA clone
Gh_D10G1415(*GhCLE77*)	ATGGCGATGCCTCACAAATCA	TTACTTGTTGGATTCAGGATT	TA clone
Gh_A11G1262(*GhCLE73*)	ATGGATATTGAACCCTTGTGT	TTACCTATTGGAAATAGGGTT	TA clone
Gh_A03G1271(*GhCLE79*)	ATGGGGGGTTCTTTGTCGTTT	CTAAGCTTGTCTTGGCGGTCG	TA clone
Gh_D12G2770(*GhCLE93*)	ATGGTTTGCAGTGCTCAAAGA	TCAAGACCTGTTGTGGATAGG	TA clone
GhCLE5A	GTCCGAGACACGTTCCGGTTGACAA	TCAATGGTGCAATGGGTTTGGACCC	Quantitative primers of GhCLE5*
S2777(Primers of arabidopsis thaliana reference genes)	GACGCTTCATCTCGTCC	CCACAGGTTGCGTTAG	Quantitative primers of GhCLE5
8991(Primers for internal reference genes of upland cotton)	CGGTGGTGTGAAGAAGCCTCAT	AATTTCACGAACAAGCCTCTGGAA	Quantitative primers of GhCLE5
pbfp4	CAAGCAATCAAGCATTCTAC	CGGACACGCTGAACTTGTGG	Universal primer for identification of GFP4 empty vector
Gh_D11G0062(GhCLE12)	tagggtacccccggggtcgacATGGCCATCAAAGTTAGCACCC	gcccttgctcaccatggatccATGGTGCAATGGGTTTGGAC	Recombinant primer for GFP vector
Gh_A11G0058(GhCLE5)	tagggtacccccggggtcgacATGGCCATCAAAGTTAGCACCC	gcccttgctcaccatggatccATGGTGCAATGGGTTTGGAC	Recombinant primer for GFP vector
Gh_A11G0197(GhCLE23)	tagggtacccccggggtcgacATGATCATCATTTGTAACCCCAAA	gcccttgctcaccatggatccGTTGTGGAGGGGGTTTGGTC	Recombinant primer for GFP vector
Gh_A08G0887(GhCLE20)	tagggtacccccggggtcgacATGTCCAGTTTCCGTGATTCCA	gcccttgctcaccatggatccGTTGTGCAGTGGGTTTGGTCC	Recombinant primer for GFP vector
Gh_A05G1794(GhCLE28	tagggtacccccggggtcgacATGATGGGAATGTTGGTACTCTCTG	gcccttgctcaccatggatccACGGTTGTGGAGTGGATCTGAG	Recombinant primer for GFP vector
Gh_A07G1675(GhCLE32)	tagggtacccccggggtcgacATGAATCGCTTTCAGCTTTGGC	gcccttgctcaccatggatccTCGCCTGTTGTGTAGTGGGTT	Recombinant primer for GFP vector
Gh_D15G1575(GhCLE39)	tagggtacccccggggtcgacATGGATCGTTTTCAGCTTTGCT	gcccttgctcaccatggatccTCGCTTGTTGTGCAAGGGG	Recombinant primer for GFP vector
Gh_D05G1490(GhCLE58)	tagggtacccccggggtcgacATGGCAATTTCGAAATTCCATC	gcccttgctcaccatggatccATGATGGATGGAGTGATGTTGGG	Recombinant primer for GFP vector
Gh_D05G1491(GhCLE59)	tagggtacccccggggtcgacATGGCTCAATGGCGATTTCTT	gcccttgctcaccatggatccGTGATGTTTGGGATCAGGGCC	Recombinant primer for GFP vector
Gh_D10G1415(GhCLE77)	tagggtacccccggggtcgacATGGCGATGCCTCACAAATC	gcccttgctcaccatggatccCTTGTTGGATTCAGGATTGGGC	Recombinant primer for GFP vector
Gh_A11G1262(GhCLE73)	tagggtacccccggggtcgacATGGATATTGAACCCTTGTGTACTCT	gcccttgctcaccatggatccCCTATTGGAAATAGGGTTTGGACC	Recombinant primer for GFP vector
Gh_A03G1271(GhCLE79)	tagggtacccccggggtcgacATGGGGGGTTCTTTGTCGTT	gcccttgctcaccatggatccAGCTTGTCTTGGCGGTCGT	Recombinant primer for GFP vector
Gh_D12G2770(GhCLE93)	tagggtacccccggggtcgacATGGTTTGCAGTGCTCAAAGAGT	gcccttgctcaccatggatccAGACCTGTTGTGGATAGGATCTGAT	Recombinant primer for GFP vector
Gh_A08G0189(GhCLE51)	tagggtacccccggggtcgacATGGCTACCTTCAATCTTTTCTTCA	gcccttgctcaccatggatccATGGTGATGAGGATCGGGACC	Recombinant primer for GFP vector
Gh_D08G0266(GhCLE62)	tagggtacccccggggtcgacATGGCTACCTTCAATGTTTCCTTC	gcccttgctcaccatggatccATGGTGATGAGGATCAGGACCA	Recombinant primer for GFP vector
Gh_D09G0020(GhCLE65)	tagggtacccccggggtcgacATGGCTAGGTTATCTTTCTTGGCT	gcccttgctcaccatggatccTGGCATTCTTGGCGGATG	Recombinant primer for GFP vector
Gh_D13G0710(GhCLE15)	tagggtacccccggggtcgacATGCTTCCCCTTCAGGCCC	gcccttgctcaccatggatccATGATGCAATGGGTTCGGAC	Recombinant primer for GFP vector
Gh_D07G0390(GhCLE10)	tagggtacccccggggtcgacATGGCCGTGAAATATACCCAGC	gcccttgctcaccatggatccATGATGCAATGGGTTTGGACC	Recombinant primer for GFP vector
Gh_A12G1391(GhCLE5)	tagggtacccccggggtcgacATGTTAAGAGGAGTGAATACAAGCG	gcccttgctcaccatggatccATTGTGAAGAGGATTAGGACCTGTATG	Recombinant primer for GFP vector
Gh_A12G1516(GhCLE83)	tagggtacccccggggtcgacATGGTTTGCAGTGCTCAAAGAGT	gcccttgctcaccatggatccAGACCTGTTGTGGATAGGATCTGAT	Recombinant primer for GFP vector
Gh_D12G2770(GhCLE93)	tagggtacccccggggtcgacATGGATATTGAACCCTTGTGGG	gcccttgctcaccatggatccCAGTCTACACACAGCTAGCTGCCC	Recombinant primer for GFP vector
GhCLE5AD	gtaccagattacgctcatatgATGGCCATCAAAGTTAGCACCC	atgcccacccgggtggaattcATGGTGCAATGGGTTTGGAC	Recombinant primers constructed by yeast vector PGADT7 and PGBKT7
CLV3AD	gtaccagattacgctcatatgATGGATTCGAAGAGTTTTCTGCTAC	atgcccacccgggtggaattcAGGGAGCTGAAAGTTGTTTCTTGG	Recombinant primers constructed by yeast vector PGADT7 and PGBKT7
CRNBD	tcagaggaggacctgcatatgATGAAGCAAAGAAGAAGAAGAAATGG	tcgacggatccccgggaattcAAAGCTGTGCAGTTGTGTAAGCA	Recombinant primers constructed by yeast vector PGADT7 and PGBKT7
CLV2BD	tcagaggaggacctgcatatgATGATAAAGATTGCAGATTTCACTCTC	tcgacggatccccgggaattcAGCTTTGGTCTGAAGAATATAACTACGA	Recombinant primers constructed by yeast vector PGADT7 and PGBKT7
CLV1BD	tcagaggaggacctgcatatgATGGCGATGAGACTTTTGAAGAC	tcgacggatccccgggaattcGAACGCGATCAAGTTCGCC	Recombinant primers constructed by yeast vector PGADT7 and PGBKT7
CLV1ECD-BD	tcagaggaggacctgcatatgTACACTGACATGGAAGTTCTTCTCAA	tcgacggatccccgggaattcCCTTGACGGTGAGAACAACGC	Recombinant primers constructed by yeast vector PGADT7 and PGBKT7
GhCLE5NLuc	acgggggacgagctcggtaccATGGCCATCAAAGTTAGCACCC	tgtagtccatttgttggatccATGGTGCAATGGGTTTGGAC	Construction of recombinant primers for Split-luciferase complementary assay
CLV3Nluc	acgggggacgagctcggtaccATGGATTCGAAGAGTTTTCTGCTAC	tgtagtccatttgttggatccTCAAGGGAGCTGAAAGTTGTTTC	Construction of recombinant primers forSplit-luciferase complementary assay
CRNCluc	tacgcgtcccggggcggtaccATGAAGCAAAGAAGAAGAAGAAATGG	tgtagtccatttgttggatccCTAAAAGCTGTGCAGTTGTGTAAGC	Construction of recombinant primers for Split-luciferase complementary assay
CLV2Cluc	tacgcgtcccggggcggtaccATGATAAAGATTGCAGATTTCACTCTC	tgtagtccatttgttggatccTTAAGCTTTGGTCTGAAGAATATAACTACG	Construction of recombinant primers for Split-luciferase complementary assay
CLV1Cluc	tacgcgtcccggggcggtaccATGGCGATGAGACTTTTGAAGAC	tgtagtccatttgttggatccTCAGAACGCGATCAAGTTCGC	Construction of recombinant primers for Split-luciferase complementary assay
GhCLE5-TRV2	gtgagtaaggttaccgaattcATGGCCATCAAAGTTAGCACCC	gtgagctcggtacctggatccGACGCCGTAACGCGGATC	Construction of recombinant primers for VIGS
GhCLE5pro	AAGTCCAACTAGCAAGCTTCGACCTCCGAGT	CAAGAGGAGAAAAACCTCAATGGTGCAATGG	promoter cloning of GhCLE5
GhCLE5pro-pBI121	tacgcagcaggtctcATCAAGAAGTCCAACTAGCAAGCTT	acccggggatcctctagaTGTTGAGGAGATTCATTAGGG	Recombinant primer for pBI121 vector in the promoter activity assay
